# Sn‐Doping‐Induced Biphasic Structure Advances Ductile Ag_2_S‐Based Thermoelectrics

**DOI:** 10.1002/advs.202408374

**Published:** 2024-09-26

**Authors:** Hao Wu, Xiao‐Lei Shi, Yuanqing Mao, Meng Li, Ting Wu, De‐Zhuang Wang, Liang‐Cao Yin, Ming Zhu, Wei‐Di Liu, Lijun Wang, Yifeng Wang, Jingui Duan, Qingfeng Liu, Zhi‐Gang Chen

**Affiliations:** ^1^ State Key Laboratory of Materials‐Oriented Chemical Engineering College of Chemical Engineering Nanjing Tech University Nanjing 211816 China; ^2^ School of Chemistry and Physics ARC Research Hub in Zero‐emission Power Generation for Carbon Neutrality and Centre for Materials Science Queensland University of Technology Brisbane Queensland 4000 Australia; ^3^ College of Materials Science and Engineering Nanjing Tech University Nanjing 211816 China

**Keywords:** Ag_2_S, interface, Sn‐doping, thermal conductivity, thermoelectric

## Abstract

Due to its inherent ductility, Ag_2_S shows promise as a flexible thermoelectric material for harnessing waste heat from diverse sources. However, its thermoelectric performance remains subpar, and existing enhancement strategies often compromise its ductility. In this study, a novel Sn‐doping‐induced biphasic structuring approach is introduced to synergistically control electron and phonon transport. Specifically, Sn‐doping is incorporated into Ag_2_S_0.7_Se_0.3_ to form a biphasic composition comprising (Ag, Sn)_2_S_0.7_Se_0.3_ as the primary phase and Ag_2_S_0.7_Se_0.3_ as the secondary phase. This biphasic configuration achieves a competitive figure‐of‐merit *ZT* of 0.42 at 343 K while retaining exceptional ductility, exceeding 90%. The dominant (Ag, Sn)_2_S_0.7_Se_0.3_ phase bolsters the initially low carrier concentration, with interfacial boundaries between the phases effectively mitigating carrier scattering and promoting carrier mobility. Consequently, the optimized power factor reaches 5 µW cm^−1^ K^−2^ at 343 K. Additionally, the formation of the biphasic structure induces diverse micro/nano defects, suppressing lattice thermal conductivity to a commendable 0.18 W m^−1^ K^−1^, thereby achieving optimized thermoelectric performance. As a result, a four‐leg in‐plane flexible thermoelectric device is fabricated, exhibiting a maximum power density of ≈49 µW cm^−2^ under the temperature difference of 30 K, much higher than that of organic‐based flexible thermoelectric devices.

## Graphical abstract

Sn‐doping is proposed to design a biphasic structure, (Ag, Sn)_2_S_0.7_Se_0.3_ as the main phase and Ag_2_S_0.7_Se_0.3_ as the interstitial phase, realizing a *ZT* of 0.42 at 343 K with >90% ductility preserved. The main phase enhances carrier concentration, while interfaces restrain carrier scattering for high mobility. Micro/nanodefects induced by the biphasic structure effectively scatter phonons, leading to low thermal conductivity.

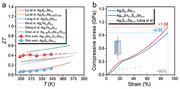

## 1 Introduction

Based on the Seebeck effect and Peltier effect, thermoelectric materials and devices can directly convert thermal energy into electrical energy and vice versa, offering a promising solution to environmental issues and resource wastage.^[^ ^1^, ^2^ ^]^ These materials and devices are typically rigid. In recent years, research on flexible thermoelectric materials and devices has emerged to effectively harness the temperature gradients generated by irregular heat sources, with ductile inorganic thermoelectric materials being a promising category.^[^ ^3^ ^]^ However, the primary challenge with these materials is their low thermoelectric performance.^[^ ^4^ ^]^ Generally, the thermoelectric performance is determined by the dimensionless figure of merit *ZT* = $\frac{S^{2}\sigma }{\kappa }$ *T*, where *S* ^2^ *σ* represents the power factor, and the parameters *S*, *σ*, *T*, and *κ* are the Seebeck coefficient, the electrical conductivity, the absolute temperature, and the total thermal conductivity, respectively.^[^ ^5^ ^]^ Additionally, the *κ* can be expressed as *κ* = *κ*_e_ + *κ*_l_, from which the parameters *κ*_e_ and *κ*_l_ are the electronic and lattice thermal conductivities, respectively.^[^ ^6^, ^7^ ^]^ To achieve the desired *ZT* values, thermoelectric materials should have high *S* ^2^ *σ* and low *κ*. However, the *S*, *σ*, and *κ*_e_ are strongly coupled with the carrier concentration *n*, and the suppression of *κ*_l_ through nanostructure engineering often leads to a decrease in *σ*, posing a significant challenge for achieving excellent thermoelectric performance in these materials.^[^ ^8^, ^9^ ^]^

As a recently discovered ductile inorganic semiconductor, α‐Ag_2_S exhibits tunable electrical transport properties and a high initial *S*, making it a promising candidate for self‐powered flexible electronics.^[^ ^4^, ^10^, ^11^, ^12^ ^]^ The exceptional ductility of Ag_2_S is due to its unique chemical bonding structure. The compound features eight‐atom ring fragments, with four silver (Ag) atoms and four sulfur (S) atoms. These ring fragments are interconnected by sulfur atoms, forming atomic layers that are stacked along the *a*‐axis.^[^ ^4^ ^]^ These multicentered chemical bonding features preserve the bonding state during slip, which prevents material cleavage. Additionally, the small energy fluctuations during slip lead to a low slip barrier, contributing to the material's exceptional ductility.^[^ ^4^ ^]^ However, pristine α‐Ag_2_S shows low thermoelectric performance (close to zero), due to its large band gap (*E*_g_) of 1–2 eV,^[^ ^13^, ^14^ ^]^ resulting in an intrinsic low *n* of 10^14^–10^17^ cm^−3^ in the near‐room temperature range, leading to low *σ*.^[^ ^15^, ^16^ ^]^ To enhance the *n* of Ag_2_S, Se/Te alloying is commonly employed to adjust the band structure and reduce the formation energy of Ag interstitials.^[^ ^17^, ^18^ ^]^ Se alloying can increase the *n* of Ag_2_S to 3.6 × 10^18^ cm^−3^, achieving a maximum *ZT* of 0.26 at room temperature while maintaining excellent ductility.^[^ ^17^, ^19^ ^]^ Te alloying introduces amorphous phases into the matrix,^[^ ^20^ ^]^ resulting in a mixed phase of amorphous and crystalline grains, giving Ag_2_S_0.4_Te_0.6_ a *ZT* of ≈0.22 at room temperature and a maximum tensile strain of ≈12.5%.^[^ ^21^ ^]^ Compared with Se or Te single‐alloying, Se/Te co‐alloying can further suppress the *κ*_l_ of Ag_2_S and achieve higher thermoelectric performance.^[^ ^22^, ^23^ ^]^ The *κ*_l_ of Ag_1.98_S_0.33_Se_0.33_Te_0.33_ alloy is reduced to ≈0.2 W m^−1^ K^−1^, leading to a high *ZT* value of 0.45 at 300 K and a high elongation of ≈60%.^[^ ^22^ ^]^ Additionally, anion site doping, such as iodine doping, has been demonstrated as an effective strategy to adjust the *n* of Ag_2_S. For instance, a maximum *ZT* of 0.26 was achieved for Ag_2_S_0.7_Se_0.295_I_0.005_ at 300 K while preserving the considerable plasticity.^[^ ^24^ ^]^

As discussed above, current research efforts primarily focus on optimizing the *n* of Ag_2_S‐based materials through Se/Te alloying and anion site doping. However, a significant challenge lies in simultaneously achieving high *ZT* and high ductility for Ag_2_S.^[^ ^25^ ^]^ Many strategies such as doping and alloying may enhance the thermoelectric performance of Ag_2_S but often result in a significant reduction in ductility.^[^ ^19^ ^]^ Currently, achieving a *ZT* above 0.4 while maintaining high ductility is extremely challenging. Therefore, conventional methods like doping and alloying to achieve a homogeneous phase structure may not be feasible. Additionally, the simultaneous attainment of high *ZT* and high ductility through multiphase design has not been reported, which is one of the key highlights of our work.

**Figure 1:**
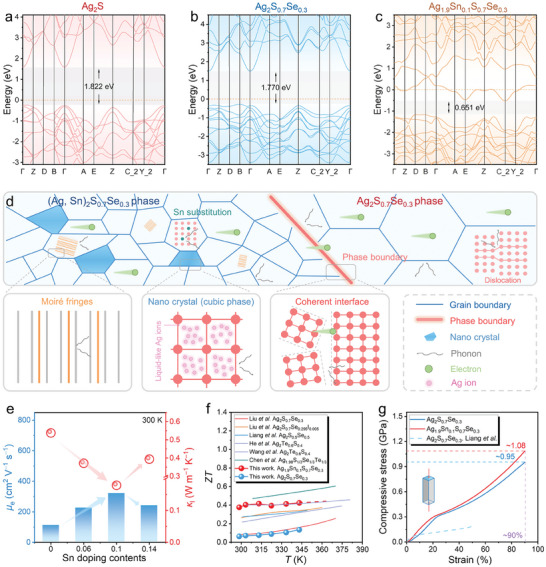
Biphasic structure design for ductile Ag_2_S‐based thermoelectric materials by Sn‐doping. Band structures of a) Ag_2_S, b) Ag_2_S_0.7_Se_0.3_, and c) Ag_1.9_Sn_0.1_S_0.7_Se_0.3_. d) Schematic diagram of the microstructural characteristics of the as‐fabricated materials. e) Electron carrier mobility *µ*_e_ and lattice thermal conductivity *κ*_l_ of Ag_2−_ *_x_* Sn*_x_* S_0.7_Se_0.3_ (*x* = 0, 0.06, 0.1, and 0.14) composites at room temperature. f) Comparison of temperature‐dependent *ZT* values of this work and reported ductile thermoelectric materials.^[^ ^17^, ^21^, ^22^, ^24^, ^26^ ^]^ g) Compressive stress–strain curves of Ag_2_S_0.7_Se_0.3_ and Ag_1.9_Sn_0.1_S_0.7_Se_0.3_ compared with that of reported work.^[^ ^17^ ^]^

To explore suitable doping elements for achieving multiphase design, in this study, we employed first‐principles density functional theory (DFT) calculations and identified tin (Sn) as an effective cationic dopant. Subsequently, we designed and fabricated a series of materials with nominal compositions Ag_2−_ *_x_* Sn*_x_* S_0.7_Se_0.3_ (*x* = 0, 0.06, 0.1, and 0.14), featuring multiple phases (Ag_2_S_0.7_Se_0.3_, (Ag, Sn)_2_S_0.7_Se_0.3_, and Ag phases) and unique microstructures. **Figure** **1**a–c illustrates the band structures of Ag_2_S, Ag_2_S_0.7_Se_0.3_, and Ag_1.9_Sn_0.1_S_0.7_Se_0.3_, respectively, calculated based on their monoclinic crystal structures (Figure S1, Supporting Information). Intrinsic Ag_2_S exhibits the *E*_g_ of ≈1.822 eV, which accounts for its inherently low *n* (≈10^14^ cm^−3^). As can be seen, Se alloying can reduce the *E*_g_ to ≈1.77 eV, slightly increasing the *n*. Sn doping at the Ag sites can further reduce the *E*_g_ to ≈0.651 eV and shift the Fermi level (*E*_F_) into the conduction band, facilitating n‐type semiconductor transport, and providing more electrons, thereby significantly enhancing the electron carrier concentration (*n*_e_) and *σ*. We compared the density of states (DOS) of Ag_2_S, Ag_2_S_0.7_Se_0.3_, and Ag_1.9_Sn_0.1_S_0.7_Se_0.3_, as shown in Figure S2 (Supporting Information), and found that Sn mainly contributes to the n‐type semiconductor behavior of Ag_1.9_Sn_0.1_S_0.7_Se_0.3_. The Snˍp orbitals near the *E*_F_ predominantly occupy the partial DOS of Sn. Furthermore, comprehensive microstructural characterizations confirmed that the prepared materials, particularly with an appropriate Sn doping concentration (*x* = 0.1), possess multi‐dimensional crystal and lattice defects, leading to a significant reduction in *κ*_l_, as depicted in Figure 1d,e. Additionally, small‐angle phase boundaries between the (Ag, Sn)_2_S_0.7_Se_0.3_ and Ag_2_S_0.7_Se_0.3_ phases ensure a high electron carrier mobility (*µ*_e_), as shown in Figure 1e. Consequently, Ag_1.9_Sn_0.1_S_0.7_Se_0.3_ achieves a peak *ZT* value of ≈0.42 at 343 K, surpassing most Ag_2_S‐based ductile semiconductors (Figure 1f).^[^ ^17^, ^21^, ^22^, ^24^, ^26^ ^]^ Meanwhile, the multiphase induced by Sn doping does not significantly compromise the plasticity, as the compressive strain of Ag_1.9_Sn_0.1_S_0.7_Se_0.3_ remains comparable to that of Ag_2_S_0.7_Se_0.3_ (Figure 1g).

## 2 Results and Discussion

**Figure 2:**
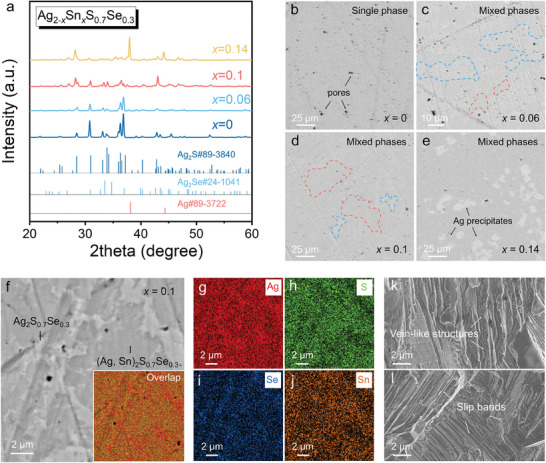
Phasic, morphological, and compositional characteristics of the Ag_2−_ *_x_* Sn*_x_* S_0.7_Se_0.3_ (*x* = 0, 0.06, 0.1, and 0.14) composites. a) X‐ray diffraction (XRD) patterns. Scanning electronic microscopy (SEM) backscattered electron (BSE) images of surface morphologies of b) Ag_2_S_0.7_Se_0.3_, c) Ag_1.94_Sn_0.06_S_0.7_Se_0.3_, d) Ag_1.9_Sn_0.1_S_0.7_Se_0.3_, and e) Ag_1.86_Sn_0.14_S_0.7_Se_0.3_ composites. f) Magnified SEM‐BSE image of Ag_1.9_Sn_0.1_S_0.7_Se_0.3_ composite. The inset shows the energy‐dispersive spectroscopy (EDS) map of overlapped elements. g–j) EDS maps of individual Ag, S, Se, and Sn elements. k,l) SEM images of fractured structures of the Ag_1.9_Sn_0.1_S_0.7_Se_0.3_.

To investigate the influence of Sn‐doping concentrations on the phase composition of Ag_2_S_0.7_Se_0.3_, a series of materials with nominal compositions Ag_2−_ *_x_* Sn*_x_* S_0.7_Se_0.3_ (*x* = 0, 0.06, 0.1, and 0.14) were prepared by using a melting method. The crystal structure and phase composition of these materials were studied by X‐ray diffraction (XRD), as shown in **Figure** **2**a. When *x* is less than 0.1, three samples exhibit similar crystal structures, namely monoclinic Ag_2_S with space group *P*21/*n*,^[^ ^17^, ^27^ ^]^ indicating successful incorporation of Sn into the Ag_2_S_0.7_Se_0.3_ lattice. All Sn‐doped samples exhibit peak broadening, indicating a decrease in grain size. The diffraction pattern of the Ag_1.86_Sn_0.14_S_0.7_Se_0.3_ (*x* = 0.14) consists of two phases, corresponding to monoclinic Ag_2_S and cubic Ag phases. To investigate the morphology and micro‐area compositional differences of the as‐prepared samples, we characterized the polished samples using scanning electron microscopy (SEM) and energy‐dispersive X‐ray spectroscopy (EDS). As shown in Figure 2b, the Ag_2_S_0.7_Se_0.3_ has a porous surface with a single phase. The Ag_1.94_Sn_0.06_S_0.7_Se_0.3_ and the Ag_1.9_Sn_0.1_S_0.7_Se_0.3_ consist of Sn‐doped (Ag, Sn)_2_S_0.7_Se_0.3_ phase (dark area) and Ag_2_S_0.7_Se_0.3_ phase (bright area), as shown in Figure 2c–e. With increasing the Sn doping concentration, the proportion of (Ag, Sn)_2_S_0.7_Se_0.3_ phase increases. In addition to the (Ag, Sn)_2_S_0.7_Se_0.3_ phase and Ag_2_S_0.7_Se_0.3_ phase, the Ag_1.86_Sn_0.14_S_0.7_Se_0.3_ contains the Ag phase, as shown in Figure 2e, which is consistent with the XRD results. The changes in phasic components across all samples with increasing Sn‐doping content can be observed in Figure S3 (Supporting Information). To obtain detailed compositions of (Ag, Sn)_2_S_0.7_Se_0.3_ and Ag_2_S_0.7_Se_0.3_ phases, we performed EDS characterization on the material with a nominal composition of Ag_1.9_Sn_0.1_S_0.7_Se_0.3_, as shown in Figure 2f–j. Detailed EDS point information is provided in Table S1 (Supporting Information), and corresponding SEM images are shown in Figure S4 (Supporting Information). The EDS point results for Ag_2_S_0.7_Se_0.3_ phase and (Ag, Sn)_2_S_0.7_Se_0.3_ phase indicate chemical formulas of Ag_2.22_S_0.56_Se_0.22_ and Ag_1.73_Sn_0.22_S_0.93_Se_0.12_, respectively. Figure 2k,l displays the vein‐like structures and slip bands of the Ag_1.9_Sn_0.1_S_0.7_Se_0.3_, which are common structural features of Ag_2_S‐based ductile materials.^[^ ^4^, ^21^, ^28^ ^]^

**Figure 3:**
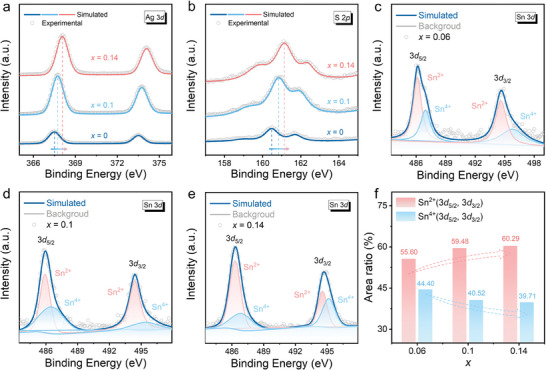
X‐ray photoelectron spectroscopy (XPS) characterizations of Ag_2−_ *_x_* Sn*_x_* S_0.7_Se_0.3_ (*x* = 0, 0.1, and 0.14) composites. High‐resolution XPS spectra of a) Ag 3*d* and b) S 2*p* for Ag_2_S_0.7_Se_0.3_, Ag_1.9_Sn_0.1_S_0.7_Se_0.3_, and Ag_1.86_Sn_0.14_S_0.7_Se_0.3_ composites. High‐resolution XPS spectra of Sn 3*d* for c) Ag_1.94_Sn_0.06_S_0.7_Se_0.3_, d) Ag_1.9_Sn_0.1_S_0.7_Se_0.3_, and e) Ag_1.86_Sn_0.14_S_0.7_Se_0.3_ composites. f) The area ratio of different oxidation states of Sn (Sn^2+^ and Sn^4+^) for Sn‐doped samples.

To investigate the valence state information of the elements in the as‐prepared samples, we utilized X‐ray photoelectron spectroscopy (XPS) characterization to study the doping mechanism of Sn. We calibrated the binding energies of Ag, Sn, and S elements by measuring the C 1*s* peak at 281.2 eV. **Figure** **3**a displays the high‐resolution XPS spectra of Ag 3*d* for Ag_2_S_0.7_Se_0.3_, Ag_1.9_Sn_0.1_S_0.7_Se_0.3_, and Ag_1.86_Sn_0.14_S_0.7_Se_0.3_ composite materials, showing significant shifts in the Ag 3*d* peak, indicating a change in the chemical environment of Ag atoms due to Sn doping.^[^ ^29^ ^]^ The doping of Sn (Sn^2+^/Sn^4+^) at Ag sites (Ag^+^) can induce the donor doping effect, providing additional electrons and altering the chemical environment of Ag atoms.^[^ ^30^ ^]^ Additionally, enlarged XPS spectra of S 2*p* for the three samples are shown in Figure 3b. The S 2*p* peak of the Sn‐doped sample shifts to higher binding energy, indicating that Sn doping weakens the average ionicity between Ag and S.^[^ ^31^ ^]^ Figure 3c–e depicts the high‐resolution XPS spectra of Sn elements for the three Sn‐doped samples, where all Sn‐doped samples exhibit both Sn^2+^ and Sn^4+^ valence states.^[^ ^30^ ^]^ Table S2 (Supporting Information) summarizes the area ratios and binding energies corresponding to Sn with different spin‐orbit coupling and oxidation states. The area ratio of Sn^2+^ increases from 55.60% for Ag_1.94_Sn_0.06_S_0.7_Se_0.3_ to 60.29% for Ag_1.86_Sn_0.14_S_0.7_Se_0.3_, while the area ratio of Sn^4+^ decreases from 44.40% to 39.71%, as shown in Figure 3f. The variation in the Sn^2+^/Sn^4+^ ratio affects the donor doping effect, thereby influencing the electrical transport properties of multiphase materials.

**Figure 4:**
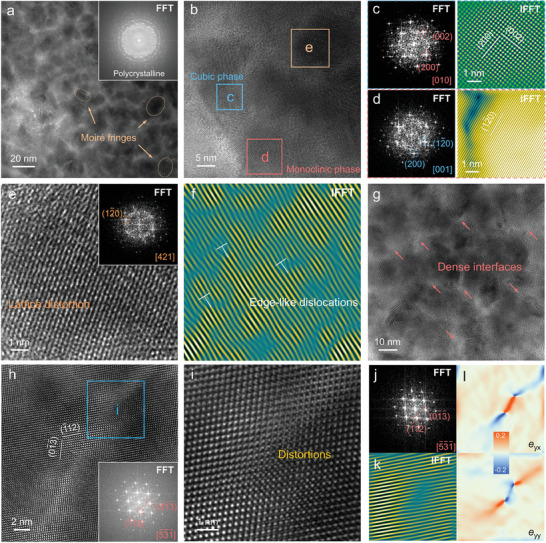
Microstructural characteristics of the Ag_1.9_Sn_0.1_S_0.7_Se_0.3_ composite. a) Transmission electron microscopy (TEM) image of the (Ag, Sn)_2_S_0.7_Se_0.3_ phase. The inset is the corresponding Fast Fourier Transform (FFT) pattern. b) High‐resolution TEM (HRTEM) image taken from of **a**. c) The FFT pattern and the inverse FFT (IFFT) pattern of the blue square area in **b**. d) The FFT pattern and IFFT pattern of the red square area in **b**. e) Enlarged HRTEM image of the yellow square in **b**. The inset is the corresponding FFT pattern. f) Corresponding IFFT pattern of **e**. g) HRTEM image of the magnified area in **a**. h) HRTEM image of the Ag_2_S_0.7_Se_0.3_ phase. The inset is the corresponding FFT pattern. i) Enlarged HRTEM image of the blue square in **h**. j) Corresponding FFT pattern of **i**. k) Corresponding IFFT pattern of **i**. l) Strain maps of **i** along different directions.

To investigate the detailed phase and microstructural characteristics, we characterized the Ag_1.9_Sn_0.1_S_0.7_Se_0.3_ using transmission electron microscopy (TEM). **Figure** **4**a depicts the TEM image of the (Ag, Sn)_2_S_0.7_Se_0.3_ phase in the Ag_1.9_Sn_0.1_S_0.7_Se_0.3_, with insets showing the corresponding fast Fourier transform (FFT) patterns. The (Ag, Sn)_2_S_0.7_Se_0.3_ phase crystallizes in a polycrystalline form, with nano‐sized grains ranging from 5 to 25 nm, consistent with the polycrystalline rings in the FFT results. Additionally, faint yellow Moiré fringes marked with ellipses can be observed within the (Ag, Sn)_2_S_0.7_Se_0.3_ phase. These Moiré fringes may stem from lattice mismatches between different sets of lattice planes and lead to local strains, as shown in Figure S5 (Supporting Information). Figure 4b presents the enlarged high‐resolution TEM (HRTEM) image of the (Ag, Sn)_2_S_0.7_Se_0.3_ phase, revealing that these nanocrystals within the (Ag, Sn)_2_S_0.7_Se_0.3_ phase crystallize into nanophases with different orientations. The FFT pattern in the blue square region of Figure 4b indicates that nanocrystals can be indexed to the [010] axis of cubic Ag_2_S, as depicted in Figure 4c. Noticeably, this cubic nanocrystalline structure is common in the (Ag, Sn)_2_S_0.7_Se_0.3_ phase, as shown in Figure S6 (Supporting Information). Cubic Ag_2_S exhibits inherently low *κ*_l_ due to its superionic properties, thus these common cubic nanocrystals can effectively scatter phonons. Figure 4d displays the FFT and IFFT patterns of the red square region in Figure 4b. The FFT pattern indicates nanocrystals pointing to the [001] axis of monoclinic Ag_2_S, with lattice planes corresponding to the (1$\bar{2}$0) plane of monoclinic Ag_2_S. Figure 4e shows the enlarged HRTEM image captured from the orange square region in Figure 4b, revealing evident lattice distortions. The corresponding IFFT pattern exhibits distinct edge‐like dislocations (Figure 4f). These dense dislocations may stem from the formation of point defects (Sn substituting Ag or Se substituting S), effectively scattering mid‐wavelength phonons. The nanoscale size of the (Ag, Sn)_2_S_0.7_Se_0.3_ phase results in dense interfaces within the phase, as depicted in Figure 4g. Figure 4h displays the HRTEM image of the Ag_2_S_0.7_Se_0.3_ phase. The pure Ag_2_S_0.7_Se_0.3_ phase without Sn exhibits a clear lattice structure, demonstrating excellent crystallinity of this phase. Such structural characteristics are entirely different from the previously described Sn‐doped (Ag, Sn)_2_S_0.7_Se_0.3_ phase. The lattice fringes correspond to the (0$\bar{1}\bar{3}$) and ($\bar{1}12$) planes of monoclinic Ag_2_S. The inset FFT pattern can be indexed along the [$\bar{5}\bar{3}\bar{1}$] axis of Ag_2_S, with another set of minor diffraction spots attributable to lattice strain, as shown in Figure S7 (Supporting Information). Figure 4i shows the enlarged HRTEM image of the selected blue square region in Figure 4h, exhibiting distinct lattice distortions. Figure 4j,k presents the corresponding FFT and IFFT patterns, confirming the existence of lattice distortions. Strain maps (*e*_yx_ and *e*_yy_) depict intense strain accompanying the distortions, with observed distortion cores, as shown in Figure 4l.

**Figure 5:**
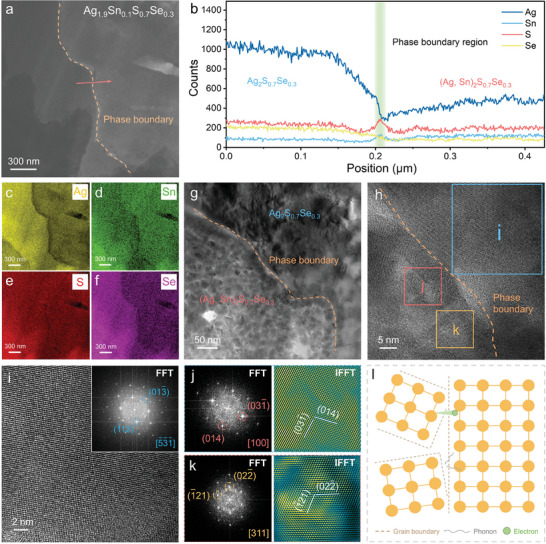
Microstructural characteristics of the phase boundaries of Ag_2_S_0.7_Se_0.3_ and Ag_1.9_Sn_0.1_S_0.7_Se_0.3_ composites. a) TEM image of the phase boundary between the Ag_2_S_0.7_Se_0.3_ phase and (Ag, Sn)_2_S_0.7_Se_0.3_ phase. b) EDS line scan along the red arrow in **a**. EDS maps of individual c) Ag, d) Sn, e) S, and f) Se elements. g) HRTEM image of the phase boundary between the Ag_2_S_0.7_Se_0.3_ phase and (Ag, Sn)_2_S_0.7_Se_0.3_ phase. h) Magnified HRTEM image of the phase boundary. i) Magnified HRTEM image of the blue square in **h**. The inset is the corresponding FFT pattern. j) Corresponding FFT pattern and IFFT pattern of the red square area in **h**. k) Corresponding FFT pattern and IFFT pattern of the yellow square area in **h**. l) Schematic diagram of the coherent phase boundary.

In addition to the internal nanostructure of the (Ag, Sn)_2_S_0.7_Se_0.3_ and Ag_2_S_0.7_Se_0.3_ phases, we conducted comprehensive HRTEM characterizations of the phase boundaries between these two phases. **Figure** **5**a illustrates the phase boundaries at low magnification in the Ag_1.9_Sn_0.1_S_0.7_Se_0.3_. Red arrow lines (EDS line scans) traverse through the Ag_2_S_0.7_Se_0.3_ phase, phase boundary, and (Ag, Sn)_2_S_0.7_Se_0.3_ phase, with the results of EDS scan lines depicted in Figure 5b. As can be seen, the Ag_2_S_0.7_Se_0.3_ phase exhibits Ag‐rich features, while the (Ag, Sn)_2_S_0.7_Se_0.3_ phase shows Sn‐rich characteristics, consistent with the SEM‐EDS results mentioned earlier. Furthermore, the concentration of Ag near the phase boundary is relatively low, as evidenced by the corresponding EDS maps of Ag, Sn, S, and Se, as shown in Figure 5c–f. To validate the atomic‐scale interface structure, HRTEM characterization was performed at the phase boundary. As depicted in Figure 5g, the (Ag, Sn)_2_S_0.7_Se_0.3_ nanophase randomly adheres to the larger Ag_2_S_0.7_Se_0.3_ phase. The magnified HRTEM image of the phase boundary is shown in Figure 5h, revealing different orientations of the (Ag, Sn)_2_S_0.7_Se_0.3_ nanophase and its epitaxial growth on the lattice planes of the Ag_2_S_0.7_Se_0.3_ phase. Figure 5i shows the HRTEM image of the blue square area in Figure 5h, the inset FFT pattern indicates that the Ag_2_S_0.7_Se_0.3_ phase can be indexed to the [$\bar{5}\bar{3}\bar{1}$] axis of monoclinic Ag_2_S, and the lattice planes correspond to the ($\bar{1}$12) plane of monoclinic Ag_2_S. The FFT and IFFT patterns in Figure 5j,k indicate that (Ag, Sn)_2_S_0.7_Se_0.3_ nanophase can be indexed to the [100] and [311] axis of monoclinic Ag_2_S, respectively. Besides, the FFT and IFFT patterns in Figure 5i,j display that the (Ag, Sn)_2_S_0.7_Se_0.3_ nanophases coherently grow on the ($\bar{1}$12) plane of Ag_2_S_0.7_Se_0.3_, with the [100] and [311] axis of (Ag, Sn)_2_S_0.7_Se_0.3_ nanophase being parallel to the [$\bar{5}\bar{3}\bar{1}$] axis of Ag_2_S_0.7_Se_0.3_ phase. Such coherent interfaces with small angles may impede phonons while barely influencing electrons,^[^ ^32^ ^]^ as illustrated in Figure 5l.

**Figure 6:**
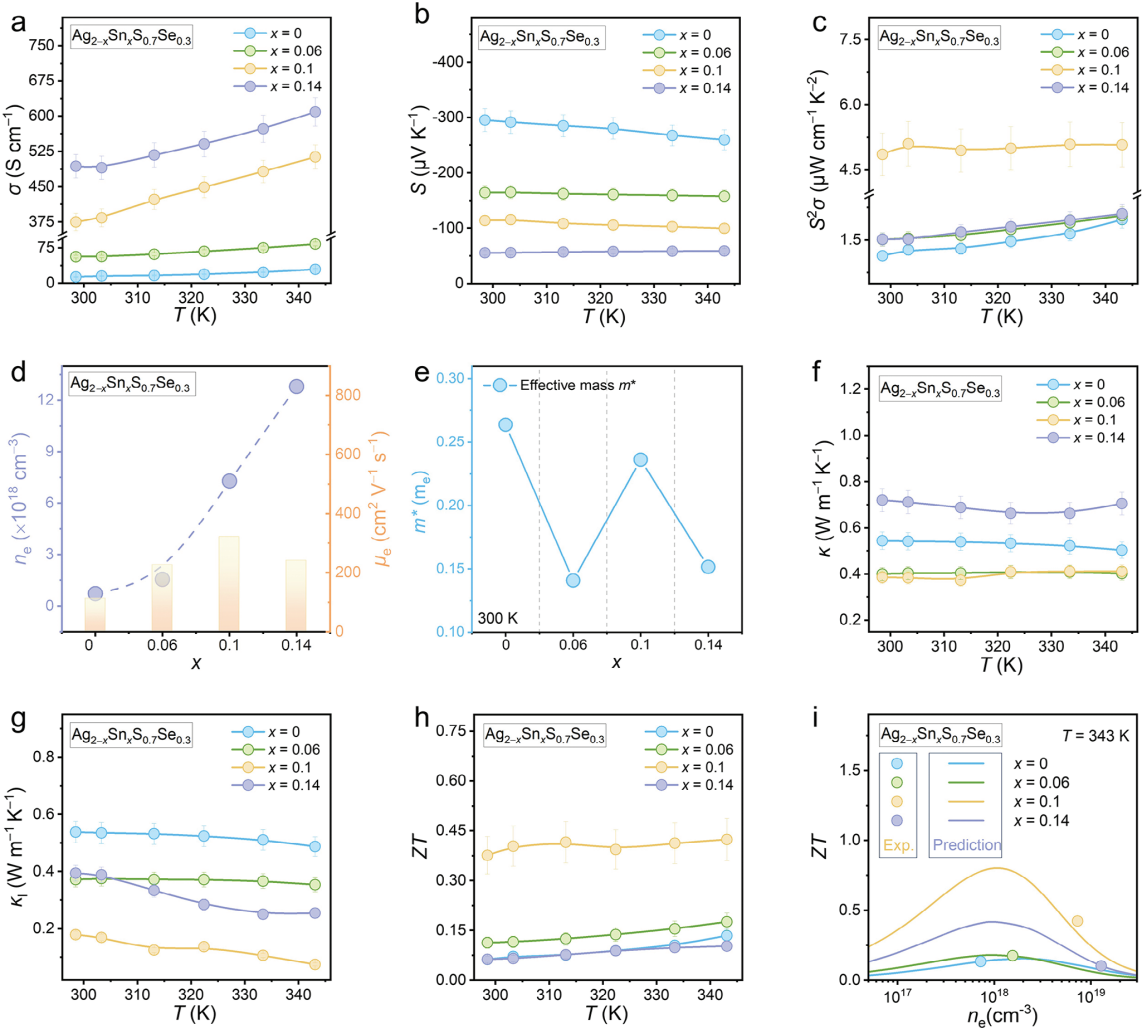
Thermoelectric properties of the Ag_2−_ *_x_* Sn*_x_* S_0.7_Se_0.3_ (*x* = 0, 0.06, 0.1, 0.14) composites. Temperature‐dependent a) electrical conductivity *σ*, b) Seebeck coefficient *S*, and c) power factor *S* ^2^ *σ*. d) *x*‐dependent electron carrier concentration *n*_e_ and *µ*_e_. e) *x*‐dependent effective mass *m* ^*^. Temperature‐dependent f) thermal conductivity *κ*, g) *κ*_l_, and h) *ZT* values. i) *n*_e_‐dependent *ZT* curves by SPB model compared with experimental data.

We measured the electrical transport properties (*n*_e_, *µ*_e_, *σ*, *S*, and *S* ^2^ *σ*) of materials with nominal compositions Ag_2−_ *_x_* Sn*_x_* S_0.7_Se_0.3_ (*x* = 0, 0.06, 0.1, and 0.14) in the temperature range of 300–343 K. **Figure** **6**a displays the *σ* of the materials, showing a significant increase with increasing Sn doping concentration. Compared to the original Ag_2_S_0.7_Se_0.3_ phase, the *σ* of Ag_1.86_Sn_0.14_S_0.7_Se_0.3_ reaches ≈500 S cm^−1^ at 300 K and ≈610 S cm^−1^ at 342 K. Furthermore, the *σ* of all samples exhibits an increasing trend with temperature, attributing to thermally excited electrons. Figure 6b presents the decreasing *S* with increasing the Sn‐doping level. Additionally, the negative values of *S* within the measured temperature range indicate that most charge carriers in these materials are electrons. Figure 6c illustrates the *S* ^2^ *σ* variation with temperature for these materials. Benefiting from the optimized *σ* and moderate *S*, the maximum *S* ^2^ *σ* of the Ag_1.9_Sn_0.1_S_0.7_Se_0.3_ increases to ≈5 µW cm^−1^ K^−2^ at 343 K, ≈2.5 times higher than the original Ag_2_S_0.7_Se_0.3_.^[^ ^17^, ^21^, ^22^, ^24^, ^26^ ^]^

To understand the changes in the electrical transport properties of the materials, the *n*_e_ and *µ*_e_ at room temperature for Ag_2−_ *_x_* Sn*_x_* S_0.7_Se_0.3_ (*x* = 0, 0.06, 0.1, and 0.14) materials are plotted in Figure 6d. The original *n*_e_ and *µ*_e_ of Ag_2_S_0.7_Se_0.3_ are ≈7.25 × 10^17^ cm^−3^ and ≈112 cm^2^ V^−1^ s^−1^, consistent with reported values.^[^ ^17^ ^]^ Doping Sn (Sn^2+^/Sn^4+^) at Ag (Ag^+^) sites can provide additional electrons to enhance *n*_e_, thus introducing (Ag, Sn)_2_S_0.7_Se_0.3_ phase in the matrix results in an increased *n*_e_ of ≈7.29 × 10^18^ cm^−3^ for Ag_1.9_Sn_0.1_S_0.7_Se_0.3_. Interestingly, the *n*_e_ enhancement induced by Sn doping does not lead to a decrease in *µ*_e_, and the *µ*_e_ of Ag_1.9_Sn_0.1_S_0.7_Se_0.3_ can reach ≈321 cm^2^ V^−1^ s^−1^. Further increasing the dopant content, the *n*_e_ of the Ag_1.86_Sn_0.14_S_0.7_Se_0.3_ increases to ≈1.28 × 10^19^ cm^−3^ due to the further increase in the intensity of (Ag, Sn)_2_S_0.7_Se_0.3_ phase and the presence of Ag phase. Such a high *n*_e_ leads to significant carrier scattering, and the additional Ag nanophase and phase boundaries considerably scatter the carriers, resulting in a decrease in the *µ*_e_ of Ag_1.86_Sn_0.14_S_0.7_Se_0.3_ to ≈241 cm^2^ V^−1^ s^−1^.

To understand the changes in *n*_e_ and *µ*_e_, we used the single parabolic band (SPB) model to calculate the effective mass (*m* ^*^) of carriers in the as‐fabricated materials, as shown in Figure 6e. Compared to Ag_2_S_0.7_Se_0.3_, the m* of Ag_1.94_Sn_0.06_S_0.7_Se_0.3_ decreases from ≈0.26 *m*_e_ to ≈0.14 *m*_e_, derived from the presence of a small amount of (Ag, Sn)_2_S_0.7_Se_0.3_ phase. The small‐angle phase boundaries have no obvious effect on filtering low‐energy carriers. For the Ag_1.9_Sn_0.1_S_0.7_Se_0.3_, with increasing Sn‐doping concentration, the content of (Ag, Sn)_2_S_0.7_Se_0.3_ phase further increases, and the *n*_e_ is greatly increased. Meanwhile, the density of phase interfaces is enhanced, thus improving the ability to filter low‐energy carriers, resulting in an overall increase in *m* ^*^ to ≈0.24 *m*_e_. However, with further increasing Sn‐doping concentration, the Ag phase begins to precipitate in the Ag_1.86_Sn_0.14_S_0.7_Se_0.3_, boosting the *n*_e_ and reducing *m* ^*^ to ≈0.15 *m*_e_ since Ag is a typical metal. Figure S8 (Supporting Information) shows the deformation potential *E*_def_. With increasing the Sn doping concentration, the *E*_def_ decreases, indicating that the deformation of the overall lattice becomes easier. This can explain why ductility can be maintained to a great extent after Sn doping.

In addition to the electrical transport properties, we also evaluated the thermal transport properties of Ag_2−_ *_x_* Sn*_x_* S_0.7_Se_0.3_ (*x* = 0, 0.06, 0.1, and 0.14), as shown in Figure 6f. For all samples, the *κ* was determined using the equation *κ* = *D*·*C*_p_·*ρ*,^[^ ^1^ ^]^ where *D* is the thermal diffusivity, *C*_p_ is the specific heat capacity, and *ρ* is the mass density. Figure S9 (Supporting Information) shows the *D* values as a function of temperature for all samples. Reasonable Sn doping can reduce the *κ* of the original Ag_2_S_0.7_Se_0.3_. Especially, the *κ* of the Ag_1.94_Sn_0.06_S_0.7_Se_0.3_ and Ag_1.9_Sn_0.1_S_0.7_Se_0.3_ is below 0.4 W m^−1^ K^−1^ at room temperature, reduced by ≈26% compared to the original Ag_2_S_0.7_Se_0.3_. Nevertheless, further increasing the Sn doping level to *x* = 0.14 leads to an increase in *κ*, ≈33% higher than the original Ag_2_S_0.7_Se_0.3_, due to the precipitation of high‐thermal‐conductivity Ag metal. To calculate the contribution of electrons to *κ*, *κ*_e_ for all samples were determined by *κ*_e_ = *σ*·*L*·*T* (Wiedemann–Franz law, where *L* is the Lorenz number),^[^ ^33^ ^]^ and *κ*_l_ was obtained by subtracting *κ*_e_. As shown in Figure S8 (Supporting Information), the *κ*_e_ of the Ag_1.86_Sn_0.14_S_0.7_Se_0.3_ increases to ≈0.45 W m^−1^ K^−1^ at 343 K, owing to the excessive *σ* induced by Sn doping and the presence of precipitated metallic Ag. Figure 6g displays the *κ*_l_ of all samples. Compared to the original Ag_2_S_0.7_Se_0.3_, the *κ*_l_ of all Sn‐doped materials was effectively reduced due to the multiscale crystals and lattice defects as described above. These defects can greatly scatter phonons of multiple wavelengths, leading to an ultra‐low room temperature *κ*_l_ of ≈0.18 W m^−1^ K^−1^ in Ag_1.9_Sn_0.1_S_0.7_Se_0.3_. Therefore, the *κ* of Ag_1.9_Sn_0.1_S_0.7_Se_0.3_ is suppressed to ≈0.4 W m^−1^ K^−1^, lower than most of reported Ag_2_S‐based thermoelectric semiconductors,^[^ ^15^, ^17^, ^20^, ^23^, ^24^, ^28^ ^]^ as shown in Figure S8 (Supporting Information). Figure 6h plots the *ZT* values of the materials, with the maximum *ZT* value of ≈0.42 at 343 K for Ag_1.9_Sn_0.1_S_0.7_Se_0.3_, ≈3 times higher than the original Ag_2_S_0.7_Se_0.3_. Such a significant enhancement can be attributed to the synergistic optimization of electrical and thermal transport properties. To evaluate the reproducibility and stability of the composite, we measured the thermoelectric performance of Ag_1.9_Sn_0.1_S_0.7_Se_0.3_ in different batches and under different heating‐cooling circles, as shown in Figures S10 and S11 (Supporting Information). Figure 6i shows the relationship between *ZT* and *n*_e_ for both experimental and calculated data by the SPB model. Evidently, by further optimizing *n*_e_ to 1 × 10^18^ cm^−3^, the Ag_1.9_Sn_0.1_S_0.7_Se_0.3_ is expected to achieve a higher *ZT* value of ≈0.8 at 343 K, competitive with traditional room temperature thermoelectric materials such as Bi_2_Te_3_ and Sb_2_Te_3_.^[^ ^34^, ^35^ ^]^

**Figure 7:**
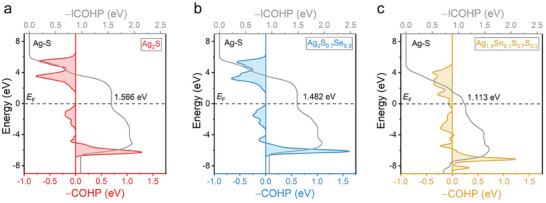
Calculated crystal orbital Hamilton population (−COHP) and integrated COHP (−ICOHP) of a) Ag_2_S, b) Ag_2_S_0.7_Se_0.3_, and c) Ag_1.9_Sn_0.1_S_0.7_Se_0.3_.

It should be noted that after Sn doping, the plasticity of the Ag_1.9_Sn_0.1_S_0.7_Se_0.3_ can be maintained at over 90% compared to the original Ag_2_S_0.7_Se_0.3_ (as shown in Figure 1g). Generally, the doping of heterogeneous atoms tends to decrease the plasticity of materials due to the diffusion strengthening effect caused by point defects induced by doping,^[^ ^36^ ^]^ leading to a significant increase in material rigidity. However, in this work, the decrease in plasticity caused by Sn doping is not significant, primarily because of the nonhomogeneous Sn doping. Sn‐doping results in the formation of the Sn‐rich (Ag, Sn)_2_S_0.7_Se_0.3_ phase, which coexists with the original Sn‐free Ag_2_S_0.7_Se_0.3_ phase. Therefore, the biphasic structure does not significantly disrupt the plasticity of the material, providing a new avenue for the design of ductile thermoelectric materials. Additionally, from the calculations, Sn‐doping does not significantly affect the plasticity of the material. The crystal orbital Hamilton population (−COHP) of Ag‐S bonding for Ag_2_S, Ag_2_S_0.7_Se_0.3_, and Ag_1.9_Sn_0.1_S_0.7_Se_0.3_ was calculated, as shown in **Figure** **7**a–c. The −COHP is utilized to depict the interaction between atomic orbitals within a crystal, thereby reflecting the nature of chemical bonds, which provides insight into the electronic structure of bonding by computing the interaction between neighboring atoms and the corresponding electron population in the orbitals. Hence, it can serve as a measure of bond strength. Generally, stronger chemical bonds result in tighter molecular interactions, potentially leading to increased hardness and brittleness of materials, while ductility may decrease. In this context, based on the computation results, pristine Ag_2_S exhibits a relatively high integrated −COHP (−ICOHP) value of 1.566 eV for Ag–S bonding. Se alloying marginally reduces this value to 1.482 eV. Additionally, Sn doping notably weakens the Ag–S bonding further, with the −ICOHP value decreasing to 1.113 eV for Ag_1.9_Sn_0.1_S_0.7_Se_0.3_, suggesting that Sn doping could potentially enhance the ductility of the materials. This result explains why the ductility of the biphasic materials can be well maintained after Sn doping.

**Figure 8:**
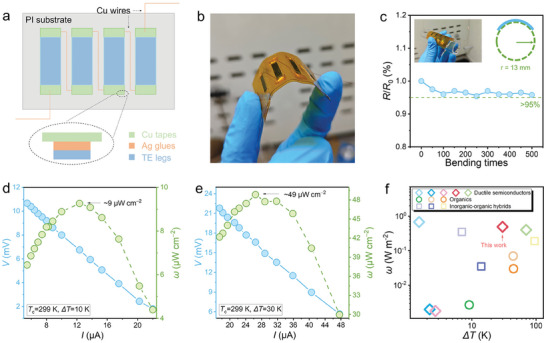
The flexible thermoelectric device and its output performance. a) The schematic diagram of an as‐fabricated in‐plane device. b) The optical graph of the device with four legs. c) Normalized resistance *R*/*R*_0_ as a function of bending times for the as‐fabricated device. The current (*I*) ‐dependent open‐circuit voltage (*V*) and power density (*ω = P*/*A = IV*/*A*) of the device under temperature difference (*ΔT*) of d) 10 K and e) 30 K. f) Comparison of *ΔT*‐dependent *ω* among ductile semiconductors, organic thermoelectrics, and inorganic‐organic hybrids.^[^ ^3^, ^28^, ^37^, ^38^, ^39^, ^40^, ^41^, ^42^, ^43^ ^]^

Considering the high thermoelectric performance and ductility of the Ag_1.9_S_0.1_S_0.7_Se_0.3_ sample, we fabricated an in‐plane flexible device consisting of a flexible polyimide (PI) substrate, Ag glues, Cu tapes, and Ag_1.9_S_0.1_S_0.7_Se_0.3_ legs, as shown in **Figure** **8**a. This configuration ensures excellent flexibility of the device, as depicted in Figure 8b. To assess its stability and flexibility further, we conducted bending tests on the device. Figure 8c illustrates the bending times‐dependent normalized resistance *R*/*R*_0_ of the devices with a bending radius of 13 mm. Over 500 bending cycles, the resistance change was less than 5%, indicating significant flexibility of the device. To verify the near‐room temperature thermoelectric performance of the material, we measured the output performance of the assembled device under different temperature differences (Δ*T*s). Figure 8d,e present the current (*I*)‐dependent output voltage (*V*) and power density (*ω*) of the in‐plane device at Δ*T*s of 10 and 30 K. Under a Δ*T* of 10 K, the device achieved a maximum *V* of ≈11 mV. Increasing Δ*T* to 30 K raised the maximum *V* to ≈22 mV, with a peak *ω* reaching ≈49 µW cm^−2^. Figure 8f summarizes the Δ*T*‐dependent *ω* of flexible thermoelectric devices, highlighting the high *ω* of our device comparable to ductile semiconductors and surpassing organic‐based thermoelectrics and inorganic‐organic hybrids, showcasing its strong potential for wearable electronic applications.

## 3 Conclusion

In summary, we theoretically and experimentally demonstrate that Sn‐doping in Ag_2_S_0.7_Se_0.3_ can induce the formation of multiphase and hierarchical microstructures. The (Ag, Sn)_2_S_0.7_Se_0.3_ phase induced by Sn‐doping exhibits higher *n*_e_ due to the additional electrons provided by Sn‐doping, which enhances the intrinsic low *n*_e_ of Ag_2_S_0.7_Se_0.3_. This allows for the synergistic optimization of the electronic and phononic behaviors of the multiphase material, leading to a peak *ZT* value of ≈0.42 at 343 K for Ag_1.9_Sn_0.1_S_0.7_Se_0.3_ material while maintaining >90% ductility. Additionally, Ag_1.9_Sn_0.1_S_0.7_Se_0.3_ features multilevel crystal and lattice defects, leading to an ultra‐low *κ*_l_ of ≈0.18 W m^−1^ K^−1^ at 300 K. Furthermore, the as‐fabricated flexible device consisting of four Ag_1.9_Sn_0.1_S_0.7_Se_0.3_ legs exhibits a peak power density of ≈49 µW cm^−2^ under the temperature difference of 30 K. Our work demonstrates that the multiphase design induced by doping can serve as an effective strategy to decouple the electrical and thermal transport properties of ductile Ag_2_S‐based semiconductors.

## Conflict of Interest

The authors declare no conflict of interest.

## Supporting information



Supporting Information

## Data Availability

The data that support the findings of this study are available from the corresponding author upon reasonable request.
