# Supplementary material for: Sn‐Doping‐Induced Biphasic Structure Advances Ductile Ag2S‐Based Thermoelectrics
Source: Adv Sci (Weinh). 2024 Sep 26;11(43):2408374. doi: 10.1002/advs.202408374 (PMC11578378; doi:10.1002/advs.202408374)
Supplement: Supplementary file 1 — Supporting Information [file ADVS-11-2408374-s001.docx]

Supporting Information

**Sn-Doping-Induced Biphasic Structure Advances Ductile Ag_2_S-Based Thermoelectrics**

*Hao Wu, Xiao-Lei Shi, Yuanqing Mao,* *Meng Li, Ting Wu, De-Zhuang Wang, Liang-Cao Yin, Ming Zhu, Wei-Di Liu, Lijun Wang,* *Yifeng Wang, Jingui Duan, Qingfeng Liu*, and* *Zhi-Gang Chen**

Mr. H. Wu, Mrs. T. Wu, Mr. D.-Z. Wang, Mr. L.-C. Yin, Mrs. M. Zhu, Prof. J. Duan, Prof. Q. Liu

State Key Laboratory of Materials-Oriented Chemical Engineering, College of Chemical Engineering, Nanjing Tech University, Nanjing 211816, China.

Email: [qfliu@njtech.edu.cn](mailto:qfliu@njtech.edu.cn)

Dr. X.-L. Shi, Mr. Y. Mao, Dr, M. Li, Dr. W.-D. Liu, Dr. L. Wang, Prof. Z.-G. Chen

School of Chemistry and Physics, ARC Research Hub in Zero-emission Power Generation for Carbon Neutrality, and Centre for Materials Science, Queensland University of Technology, Brisbane, Queensland 4000, Australia.

E-mail: zhigang.chen@qut.edu.au

Prof. Y. Wang

College of Materials Science and Engineering, Nanjing Tech University, Nanjing 211816, China.

Keywords: thermoelectric, Ag_2_S, Sn-doping, interface, thermal conductivity.

**Experimental section**

*Materials synthesis*. Ag_2−_*_x_*Sn*_x_*S_0.7_Se_0.3_ (*x* = 0, 0.06, 0.1, 0.14) samples were fabricated using a melting process from high-purity elemental Ag, Sn, S, and Se (99.99%, China National Building Material (CNBM, Chengdu) Optoelectronic Materials Co., Ltd.). Stoichiometric amounts of Ag, Sn, S, and Se were sealed in quartz tubes under high vacuum (~10^−3^ Pa). The tubes were then heated to 1223 K over 10 hours, held at this temperature for 12 hours, and quenched in ice water. Subsequently, the tubes were annealed at 723 K for 2 days. The obtained ingots were cut into pieces of various dimensions. The relative densities of all synthesized samples exceeded 98 %.

*Characterization*. The structural characteristics of Ag_2−_*_x_*Sn*_x_*S_0.7_Se_0.3_ (*x* = 0, 0.06, 0.1, 0.14) samples were investigated using X-ray diffraction (XRD) analysis performed on a Smart-lab 3 KW instrument with a Cu source. The microstructure of the Ag_1.9_Sn_0.1_S_0.7_Se_0.3_ composite was examined using field emission scanning electron microscopy (FE-SEM) on a JEOL-S4800 instrument and tabletop optical microscopy on a Hitachi TM3000, coupled with X-ray energy-dispersive spectroscopy (EDS) using OXFORD Xplore. The chemical states of all samples were analyzed using X-ray photoelectron spectroscopy (XPS) on a Thermo ESCALAB 250 instrument. Furthermore, the nanostructural features of all composites were observed using probe-corrected scanning transmission electron microscopy (STEM) equipped with Silicon Drift Detector detectors operated at 200 KeV, along with EDS detectors.

*Performance evaluation*. The electrical transport properties (conductivity *σ* and Seebeck coefficient *S*) were assessed using an SBA-458 instrument from NETZSCH, Germany. The uncertainty in the measurements of *σ* and *S* was determined to be 5 % and 7 %, respectively. Thermal diffusivity (*D*) was determined *via* the laser flash method using an LFA-457 instrument, also from NETZSCH, Germany, with an uncertainty of 7 %. The specific heat (*C*_p_) was calculated using the Dulong-Petit law, yielding a *C*_p_ of 0.276 J g^−1^ K^−1^ for the Ag_2_S_0.7_Se_0.3_ sample. Sn doping at Ag-sites can reduce the *C*_p_ of Ag_2_S_0.7_Se_0.3_ due to the larger relative atomic mass of Sn, resulting in a lower *C*_p_. Additionally, the *C*_p_ value of Ag precipitate is 0.24 J g^−1^ K^−1^. Since the exact fractions of Ag_2_S_0.7_Se_0.3_, (Ag, Sn)_2_S_0.7_Se_0.3_, and Ag precipitate were not determined, the *C*_p_ of 0.276 J g^−1^ K^−1^ was assumed for all samples to prevent overestimating thermal transport properties. The total thermal conductivity (*κ*) was determined using the formula *κ* = *D*·*C*_p_·*ρ*, with an uncertainty of approximately 7 %. The combined uncertainty in the calculated *ZT* was 15 %. The densities of all samples were measured using the Archimedes method.^[1]^ The Hall coefficient (*R*) was measured using the Van der Pauw method with a CH-70 instrument from CH-magnetoelectricity Technology Co., Ltd., China, under magnetic fields up to 500 mT. Carrier concentration (*n*) and mobility (*μ*) were determined using the equations *n* = 1⁄*eR* and *μ* = *σR*, respectively. After assessing the thermoelectric performances, the desk samples were cut into cuboids using a diamond saw to measure mechanical properties. The compression tests were conducted using an INSTRON 5982 universal machine with a loading rate of 0.5 mm min^−1^.

*Device fabrication and test*. A four-leg device was constructed using four pieces of 20 mm × 3 mm × 0.075 mm Ag_1.9_S_0.1_S_0.7_Se_0.3_ thin foils, which were cold pressed and rolled to reduce thickness. After cold pressing and rolling, Ag_1.9_Sn_0.1_S_0.7_Se_0.3_ film possesses different electrical transport properties compared to the bulk. Therefore, the experimental value of the open circuit voltage (*V*_oc_) is different from the theoretical value calculated based on Ag_1.9_Sn_0.1_S_0.7_Se_0.3_ bulk, as illustrated in **Figure S12**. Polyimide (PI) substrates served as support bases, with thermoelectric legs attached using adhesive tapes. Interconnections between the four legs were established using 0.2 mm diameter Cu wires. Conductive Cu tapes functioned as electrodes, connected to thermoelectric legs with Ag glues. The device internal resistance is around 79 Ω, which is close to the theoretical resistance of about 77.5 Ω. The contact resistance of the device is approximately 1.5 Ω. A custom-built testing instrument equipped with a source meter (K 2450, Keithley) was used to measure the device output performance using the 4-wire method. Temperature difference (Δ*T*) was controlled using a heater and a lab-made radiator, with measurement conducted *via* two thermocouples. The cold side was maintained at 26 °C. *I*-*V* curves of the devices under different Δ*T*s were recorded. The output power density of the device was calculated using *ω* = *IV*/*A*.

*Calculation.* First-principles calculations were performed based on density-functional theory (DFT) with all electron projected augmented wave (PAW) method, as implemented in the Vienna Ab initio Simulation Package (VASP).^[2-7]^ Semi-local generalized gradient approximation (GGA) with the fully relativistic Perdew-Burke-Ernzerhof (PBE) exchange correlation functional was employed.^[8]^ The Brillouin zone was sampled by a Monkhorst-Pack **k**-mesh spanning less than 0.03/Å^3^ for structural relaxation, and a denser **k**-mesh spanning less than 0.015/Å^3^ for non-self-consistent calculations. The wave functions were expanded on a plan-wave basis with a cut-off energy of 450 eV. All atoms were allowed to relax in their geometric optimizations until the Hellmann–Feynman force is less than 1×10^–3^ eV·Å^–1^, and the convergence criterion for the electronic self-consistent loop was set to 1×10^–7^ eV. The electron band structures were calculated along the line-mode **k**-path based on Brillouin path features indicated by the AFLOW framework.^[9]^ To precisely calculate the bandgap, the combination of the modified Becke-Johnson (MBJ) method and an effective onsite coulombic U (the U value for Ag_4d orbital is determined to be 5.8 eV by using the linear corresponding method) was adopted.^[10-12]^ The LOBSTER package was utilized to reveal the bonding characteristics partially indicating the bonding strength.^[13]^

*Modelling*. For calculation details of the single parabolic band (SPB) modeling, there are:^[9, 14-17]^

$S\left( \eta\right)=\frac{k_{B}}{e}\cdot\left[ \frac{\left( g+\frac{5}{2} \right)\cdot F_{g+\frac{3}{2}}\left( \eta\right)}{\left( g+\frac{3}{2} \right)\cdot F_{g+\frac{1}{2}}\left( \eta\right)}-\eta\right]$ (S1-1)

$n=\frac{1}{e\cdot R_{H}}=\frac{{(2m^{*}\cdot k_{B}T)}^{\frac{3}{2}}}{3\pi^{2}\hbar^{3}}\cdot\frac{\left( g+\frac{3}{2} \right)^{2}\cdot F_{g+\frac{1}{2}}^{2}(\eta)}{(2g+\frac{3}{2})\cdot F_{2g+\frac{1}{2}}(\eta)}$ (S1-2)

$\mu=\left[ \frac{e\pi\hbar^{4}}{\sqrt{2}{(k_{B}T)}^{\frac{3}{2}}}\frac{C_{l}}{{E_{\mathrm{def}}}^{2}{(m^{*})}^{\frac{5}{2}}} \right]\frac{(2g+\frac{3}{2})\cdot F_{2g+\frac{1}{2}}(\eta)}{\left( g+\frac{3}{2} \right)^{2}\cdot F_{g+\frac{1}{2}}(\eta)}$ (S1-3)

$L={(\frac{k_{B}}{e})}^{2}\cdot\left\{ \frac{\left( g+\frac{7}{2} \right)\cdot F_{g+\frac{5}{2}}\left( \eta\right)}{\left( g+\frac{3}{2} \right)\cdot F_{g+\frac{1}{2}}\left( \eta\right)}-\left[ \frac{\left( g+\frac{5}{2} \right)\cdot F_{g+\frac{3}{2}}\left( \eta\right)}{\left( g+\frac{3}{2} \right)\cdot F_{g+\frac{1}{2}}\left( \eta\right)} \right]^{2} \right\}$ (S1-4)

where *η* is the reduced Fermi level, *k*_B_ is the Boltzmann constant, *g* is the carrier scattering factor (*g* = -1/2 for acoustic phonon scattering), *ħ* is the reduced plank constant, *C*_l_ is the elastic constant for longitudinal vibrations, *E*_def_ is the deformation potential coefficient, *m** is the effective mass, and *L* is the Lorenz number. For *C*_l_, there is:^[14-17]^

*C*_l_ *= v*_l_*^2^⋅ρ* (S1-5)

where *v*_l_ is the longitudinal sound velocity of ~2596 m s^−1^ as measured.^[18]^ *F_i_(η)* is the Fermi integral expressed as:^[14-17]^

$F_{i}\left( \eta\right)=\int_{0}^{\infty} \frac{x^{i}}{1+e^{(x-\eta)}}dx$ (S1-6)

**Table S1.** Energy-dispersive spectroscopy (EDS) results of Ag_1.9_Sn_0.1_S_0.7_Se_0.3_ composite.

|  | **#1** | **#2** | **#3** | **#4** | **#5** | **Average** |
| --- | --- | --- | --- | --- | --- | --- |
| Ag (at%) | 58.12 | 58.43 | 58.05 | 58.47 | 58.67 | 58.35 |
| S (at%) | 29.95 | 30.56 | 29.92 | 30.53 | 30.51 | 30.29 |
| Se (at%) | 4.52 | 3.90 | 4.81 | 3.80 | 3.77 | 4.16 |
| Sn (at%) | 7.41 | 7.11 | 7.22 | 7.20 | 7.05 | 7.20 |
|  | **#6** | **#7** | **#8** | **#9** | **#10** | **Average** |
| Ag (at%) | 55.62 | 58.79 | 58.35 | 56.97 | 58.43 | 57.63 |
| S (at%) | 32.17 | 30.67 | 30.32 | 31.38 | 30.91 | 31.09 |
| Se (at%) | 4.21 | 3.81 | 4.21 | 4.04 | 3.50 | 3.95 |
| Sn (at%) | 8.00 | 6.74 | 7.12 | 7.82 | 7.16 | 7.37 |
|  | **#11** | **#12** | **#13** | **#14** | **#15** | **Average** |
| Ag (at%) | 73.92 | 73.42 | 74.28 | 74.63 | 74.32 | 74.11 |
| S (at%) | 18.68 | 18.42 | 18.20 | 18.06 | 18.10 | 18.29 |
| Se (at%) | 7.40 | 8.16 | 7.52 | 7.31 | 7.58 | 7.59 |
|  | **#16** | **#17** | **#18** | **#19** | **#20** | **Average** |
| Ag (at%) | 73.57 | 73.82 | 74.64 | 74.56 | 73.78 | 74.07 |
| S (at%) | 18.88 | 18.91 | 17.95 | 18.17 | 18.44 | 18.47 |
| Se (at%) | 7.55 | 7.27 | 7.41 | 7.27 | 7.78 | 7.46 |

**Table S2.** Binding energy and percentage area obtained by deconvolution of X-ray Photoelectron Spectroscopy (XPS) spectra for different oxidation states of Sn.

| **Composition** | **Oxidation state** | **Area (%)** | **Spin orbit** | **Binding energy (eV)** |
| --- | --- | --- | --- | --- |
| *x* = 0.06 | Sn^2+^ | 55.60 | Sn 3*d*_5/2_ | 486.1 |
|  |  |  | Sn 3*d*_3/2_ | 494.6 |
|  | Sn^4+^ | 44.40 | Sn 3*d*_5/2_ | 486.8 |
|  |  |  | Sn 3*d*_3/2_ | 496.6 |
| *x* = 0.1 | Sn^2+^ | 59.48 | Sn 3*d*_5/2_ | 485.9 |
|  |  |  | Sn 3*d*_3/2_ | 494.4 |
|  | Sn^4+^ | 40.52 | Sn 3*d*_5/2_ | 486.6 |
|  |  |  | Sn 3*d*_3/2_ | 495.4 |
| *x* = 0.14 | Sn^2+^ | 60.29 | Sn 3*d*_5/2_ | 486.3 |
|  |  |  | Sn 3*d*_3/2_ | 494.6 |
|  | Sn^4+^ | 39.71 | Sn 3*d*_5/2_ | 486.9 |
|  |  |  | Sn 3*d*_3/2_ | 495.2 |

**Table S3.** The actual density *ρ*, theoretical density *ρ*_0_, and relative density values of Ag_2−_*_x_*Sn*_x_*S_0.7_Se_0.3_ (*x* = 0, 0.06, 0.1, 0.14) samples.

| Composition | 0 | 0.06 | 0.1 | 0.14 |
| --- | --- | --- | --- | --- |
| *ρ* | 7.52 | 7.50 | 7.49 | 7.45 |
| *ρ*_0_ | 7.60 | 7.60 | 7.60 | 7.60 |
| % | 98.9 | 98.7 | 98.6 | 98.0 |


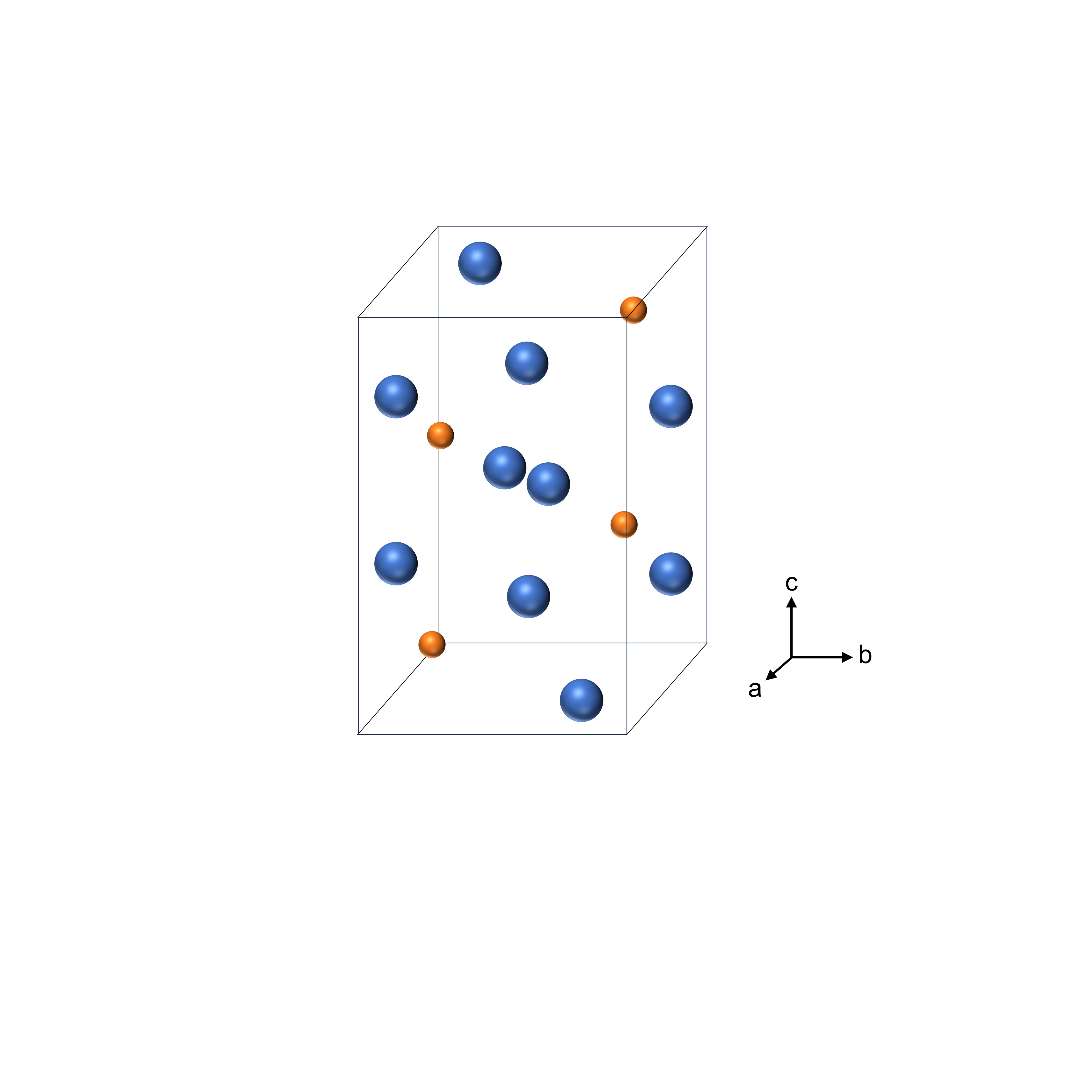


**Figure S1.** Unit cell of monoclinic Ag_2_S.


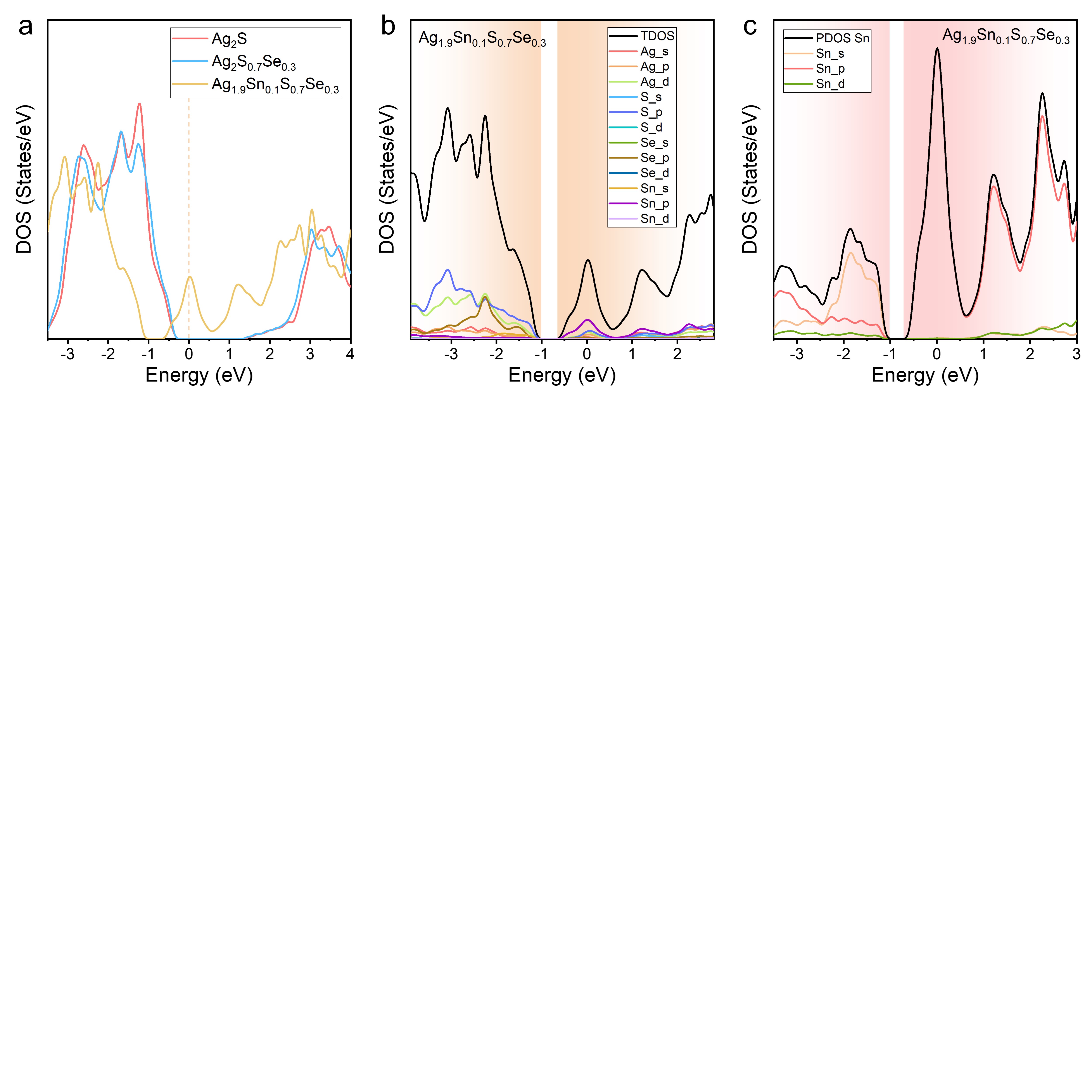


**Figure S2.** a) Density of states (DOS) of Ag_2_S, Ag_2_S_0.7_Se_0.3_, and Ag_1.9_Sn_0.1_S_0.7_Se_0.3_. b) DOS of Ag_1.9_Sn_0.1_S_0.7_Se_0.3_. c) Partial DOS of Sn for Ag_1.9_Sn_0.1_S_0.7_Se_0.3_.


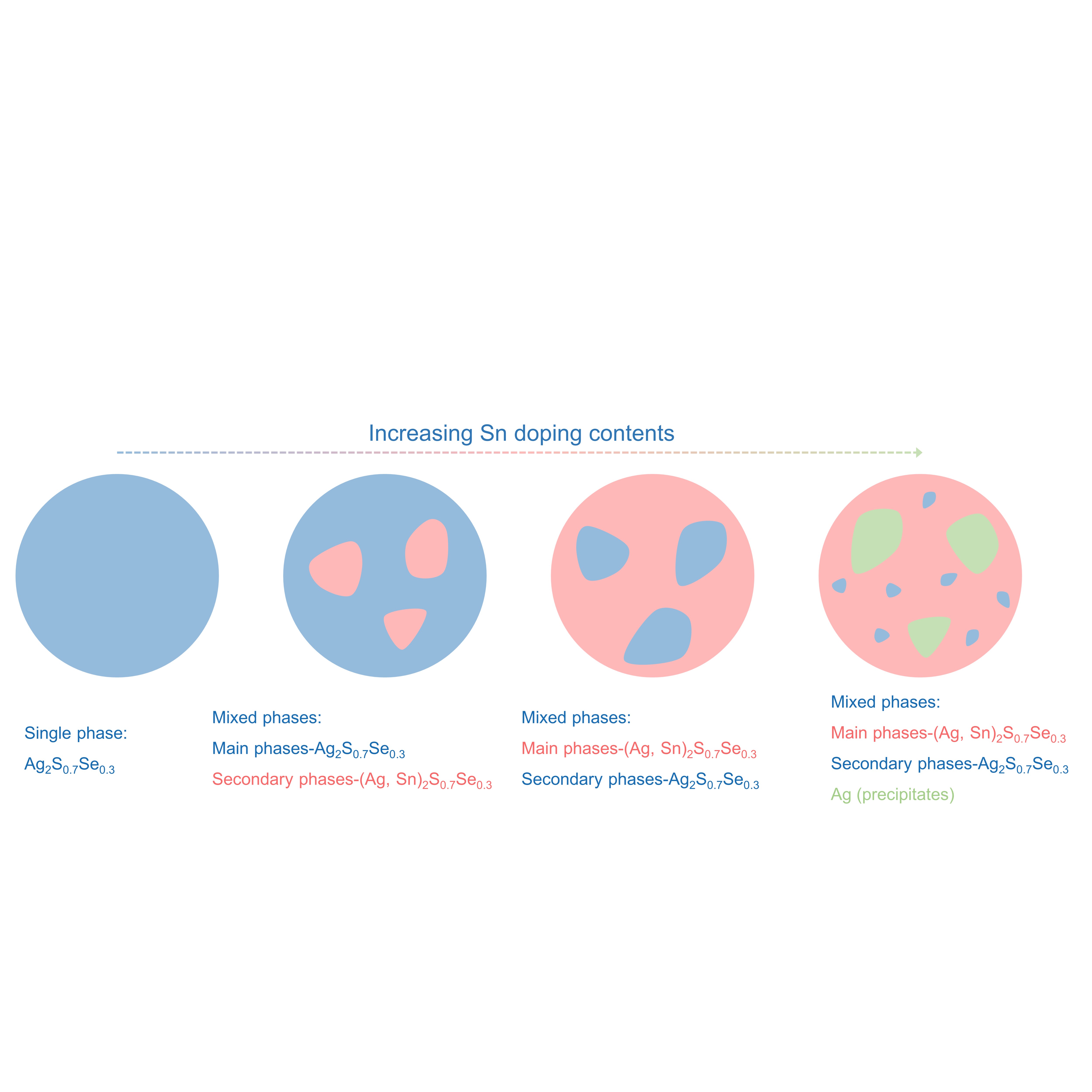


**Figure S3.** Schematic diagram of phasic components of Ag_2_S_0.7_Se_0.3_, Ag_1.94_Sn_0.06_S_0.7_Se_0.3_, Ag_1.9_Sn_0.1_S_0.7_Se_0.3_, and Ag_1.86_Sn_0.14_S_0.7_Se_0.3_.


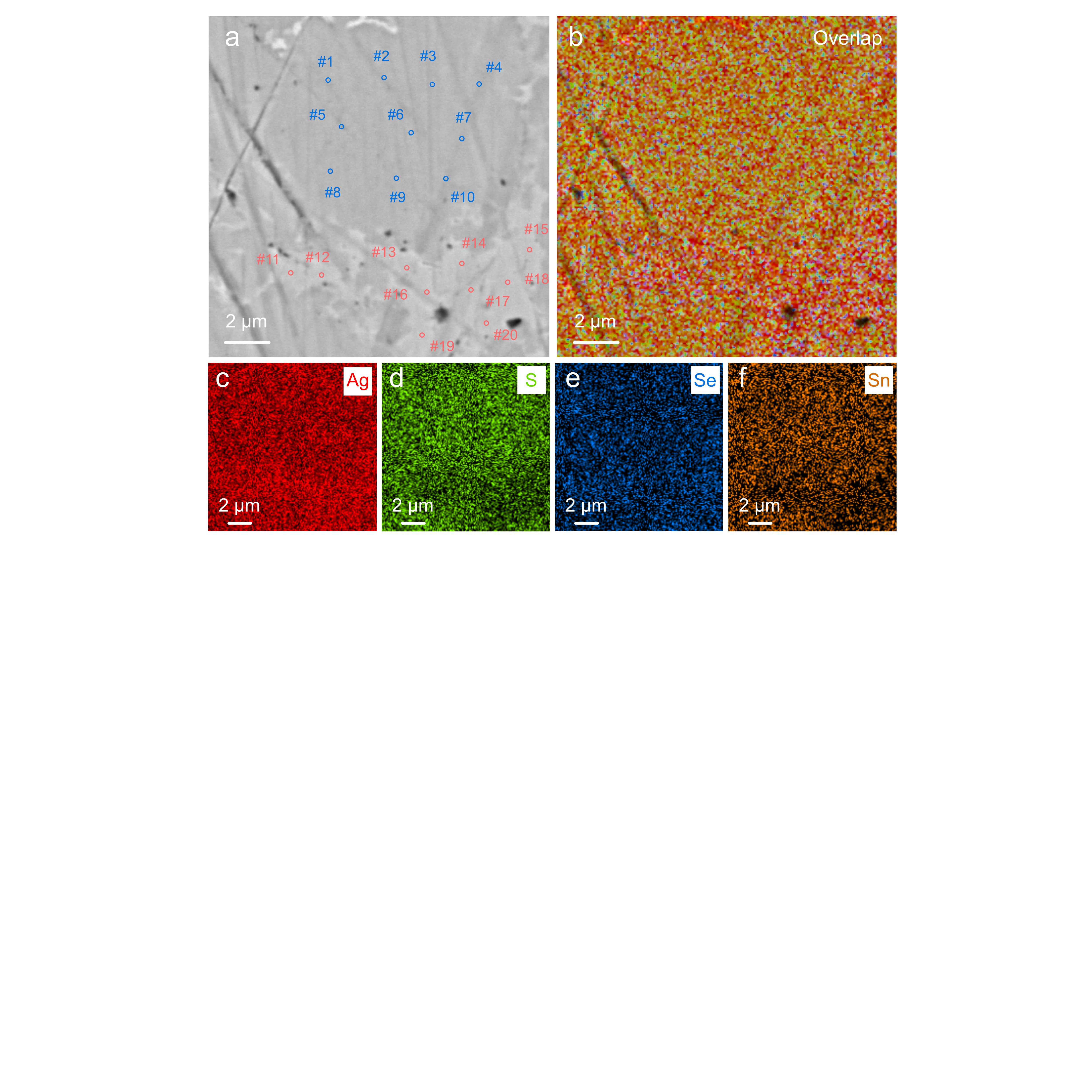


**Figure S4.** a) Scanning electronic microscopy (SEM) back scattered electron (BSE) image of Ag_1.9_Sn_0.1_S_0.7_Se_0.3_ composite. Energy-dispersive spectroscopy (EDS) maps for b) overlap of Ag, S, Se, and Sn. EDS maps of individual c) Ag, d) S, e) Se, and f) Sn elements.

**
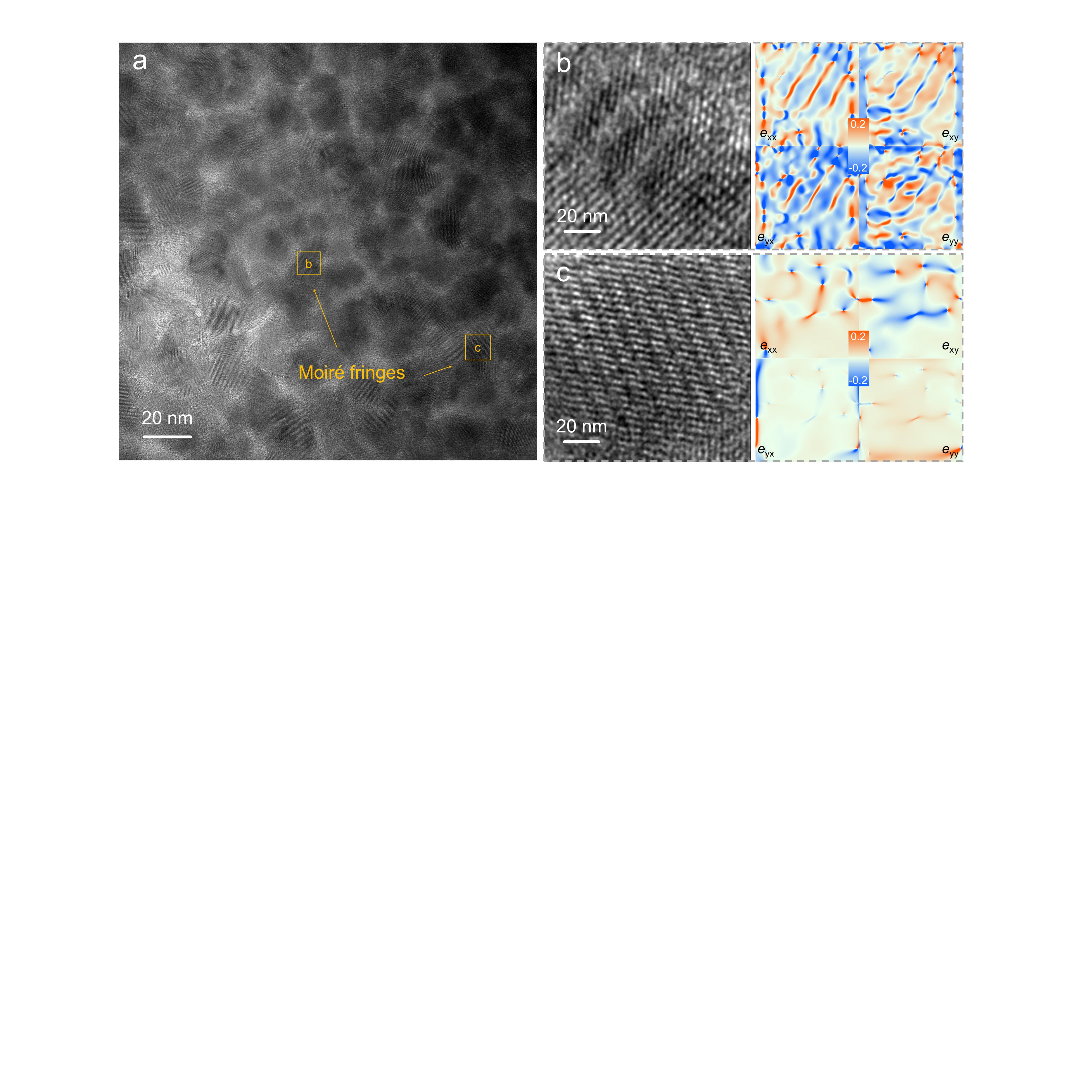
**

**Figure S5.** a) High-resolution transmission electron microscopy (HRTEM) image of the (Ag, Sn)_2_S_0.7_Se_0.3_ phase. b, c) Enlarged HRTEM images of the orange square areas in **a** and the corresponding strain maps along different directions.

**
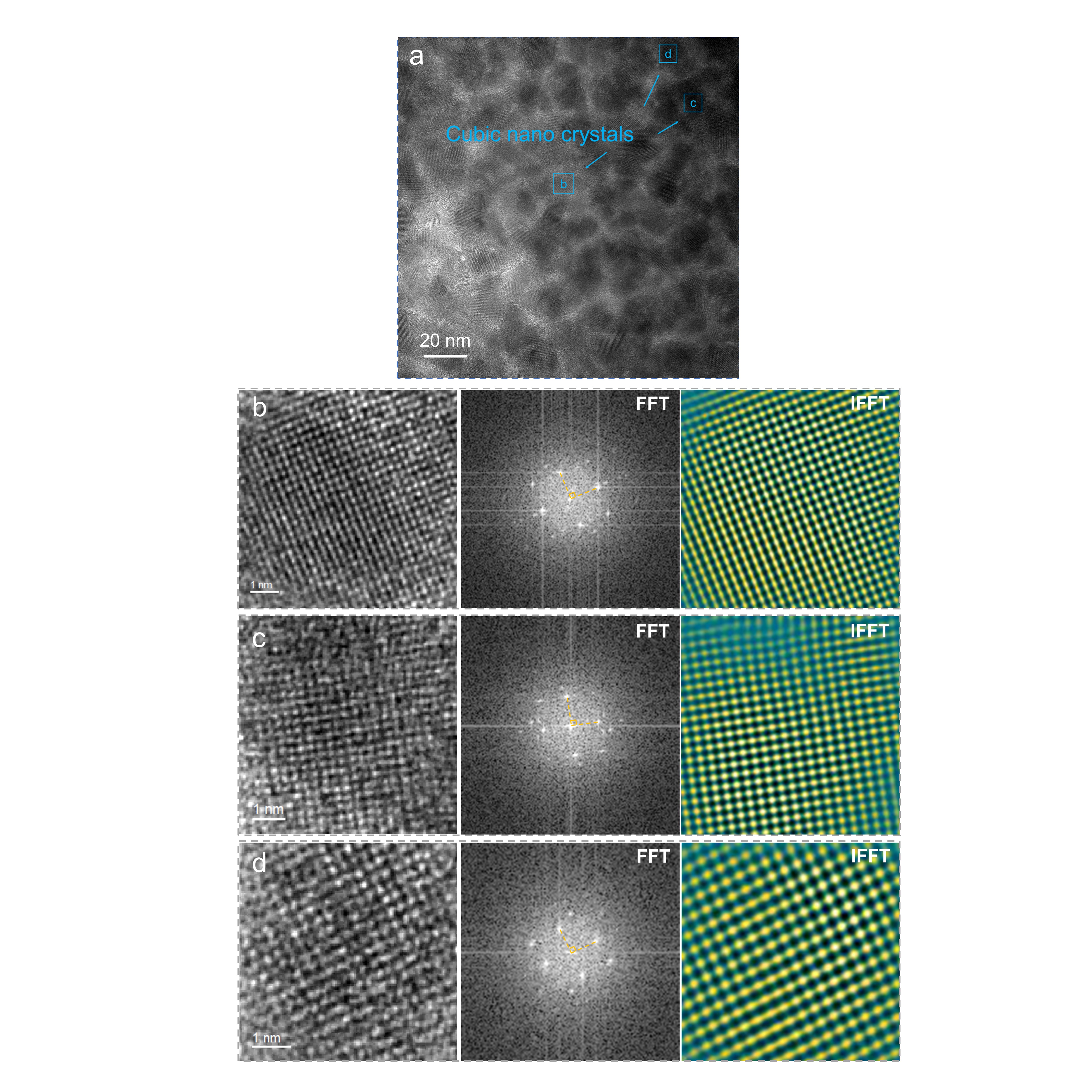
**

**Figure S6.** a) HRTEM image of the (Ag, Sn)_2_S_0.7_Se_0.3_ phase. b, c, d) Enlarged HTEM images of the blue square areas in **a** and the corresponding Fast Fourier Transform (FFT) and inverse FFT (IFFT) images.


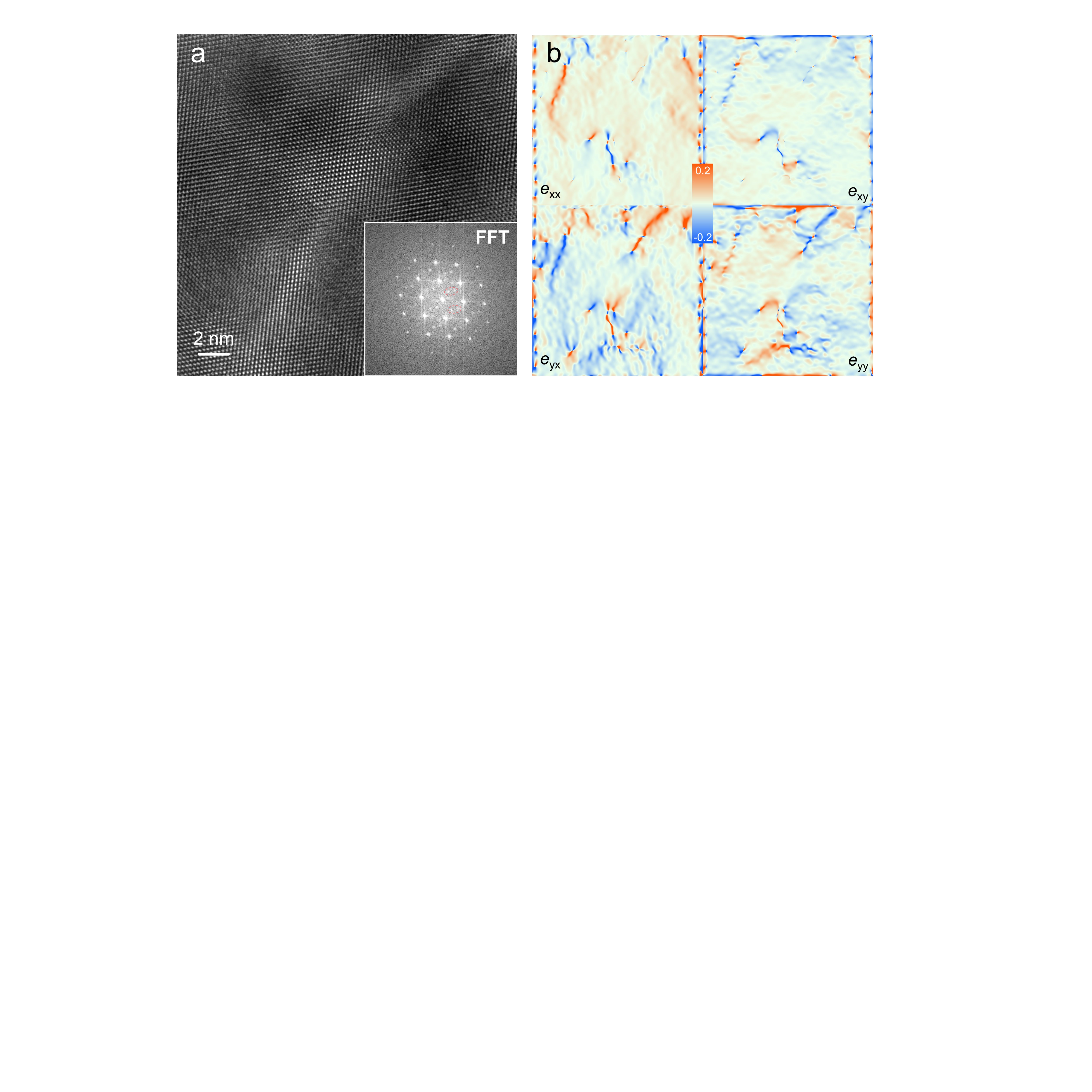


**Figure S7.** a) HRTEM image of the Ag_2_S_0.7_Se_0.3_ phase. The inset is the corresponding FFT pattern. b) Corresponding strain maps.

**
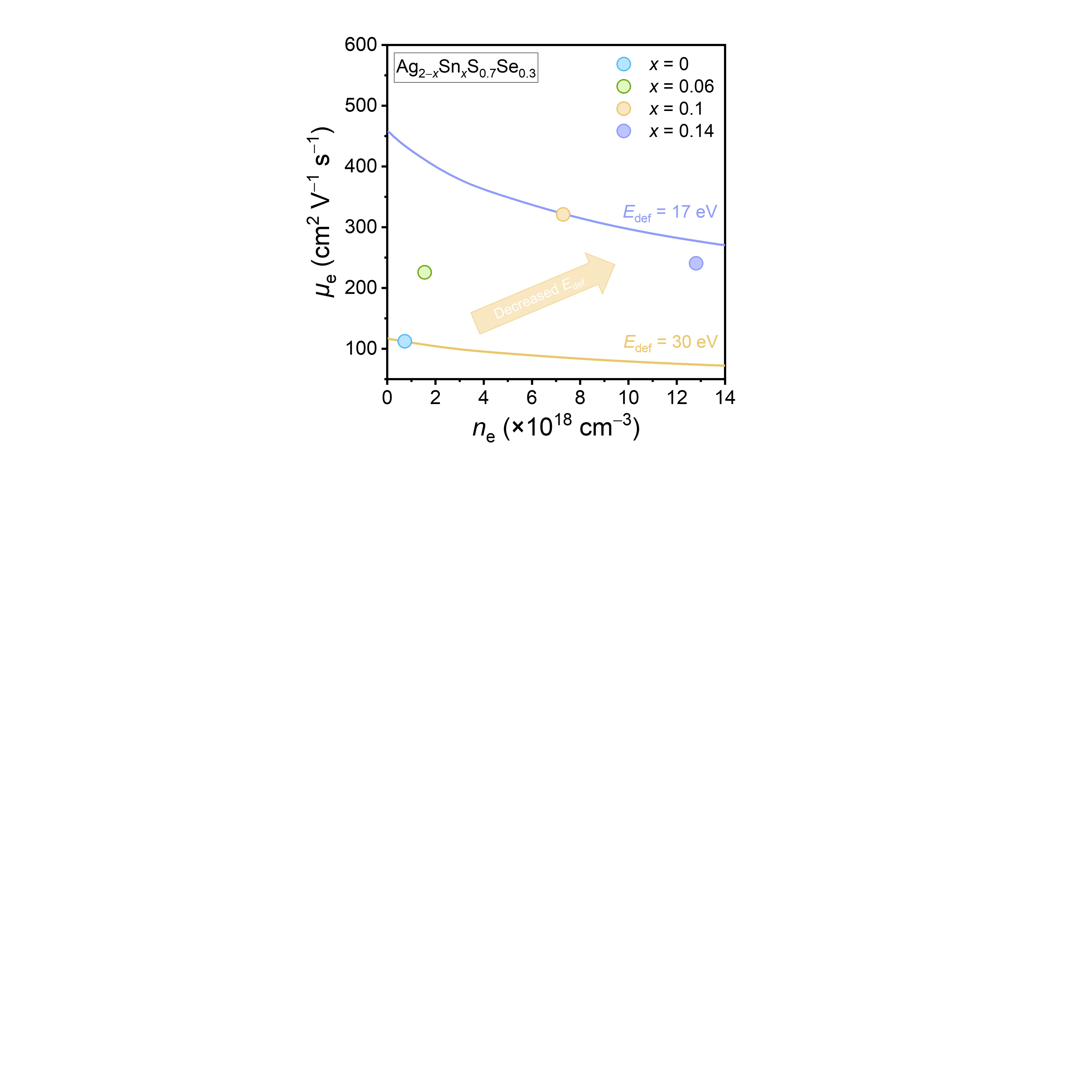
**

**Figure S8.** The electron carrier concentration (*n*_e_)-dependent electron carrier mobility (*μ*_e_) of Ag_2−_*_x_*Sn*_x_*S_0.7_Se_0.3_ (*x* = 0, 0.06, 0.1, 0.14) composites at 300 K. The inset lines represent the theoretical curves based on single parabolic band model.

**
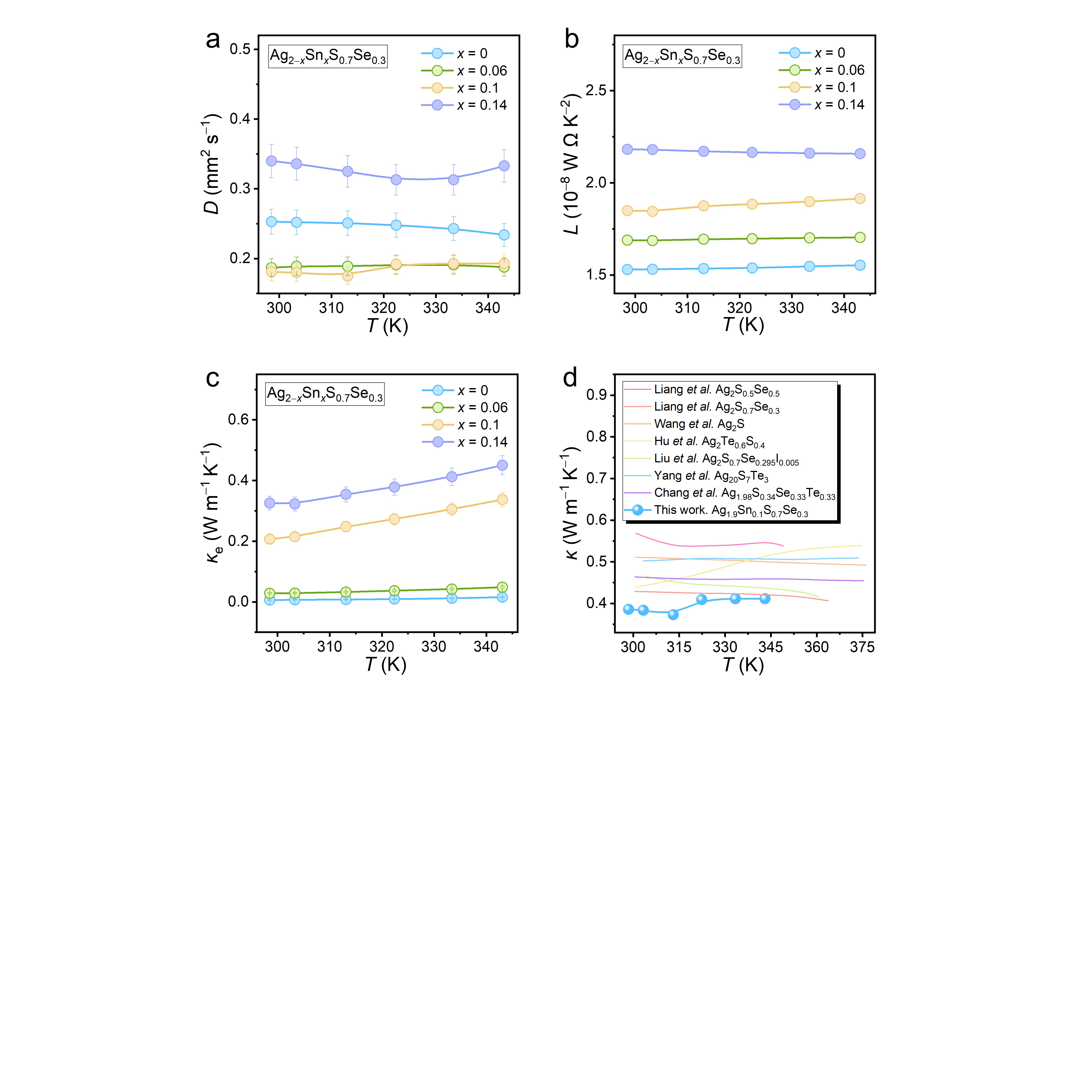
**

**Figure S9.** Temperature-dependent thermal transport properties of Ag_2−_*_x_*Sn*_x_*S_0.7_Se_0.3_ (*x* = 0, 0.06, 0.1, 0.14) composites. Temperature-dependent a) thermal diffusivity (*D*), b) Lorenz number (*L*), and c) electronic thermal conductivity (*κ*_e_). d) Summary of thermal conductivity (*κ*) of ductile thermoelectric materials.^[18-23]^

**
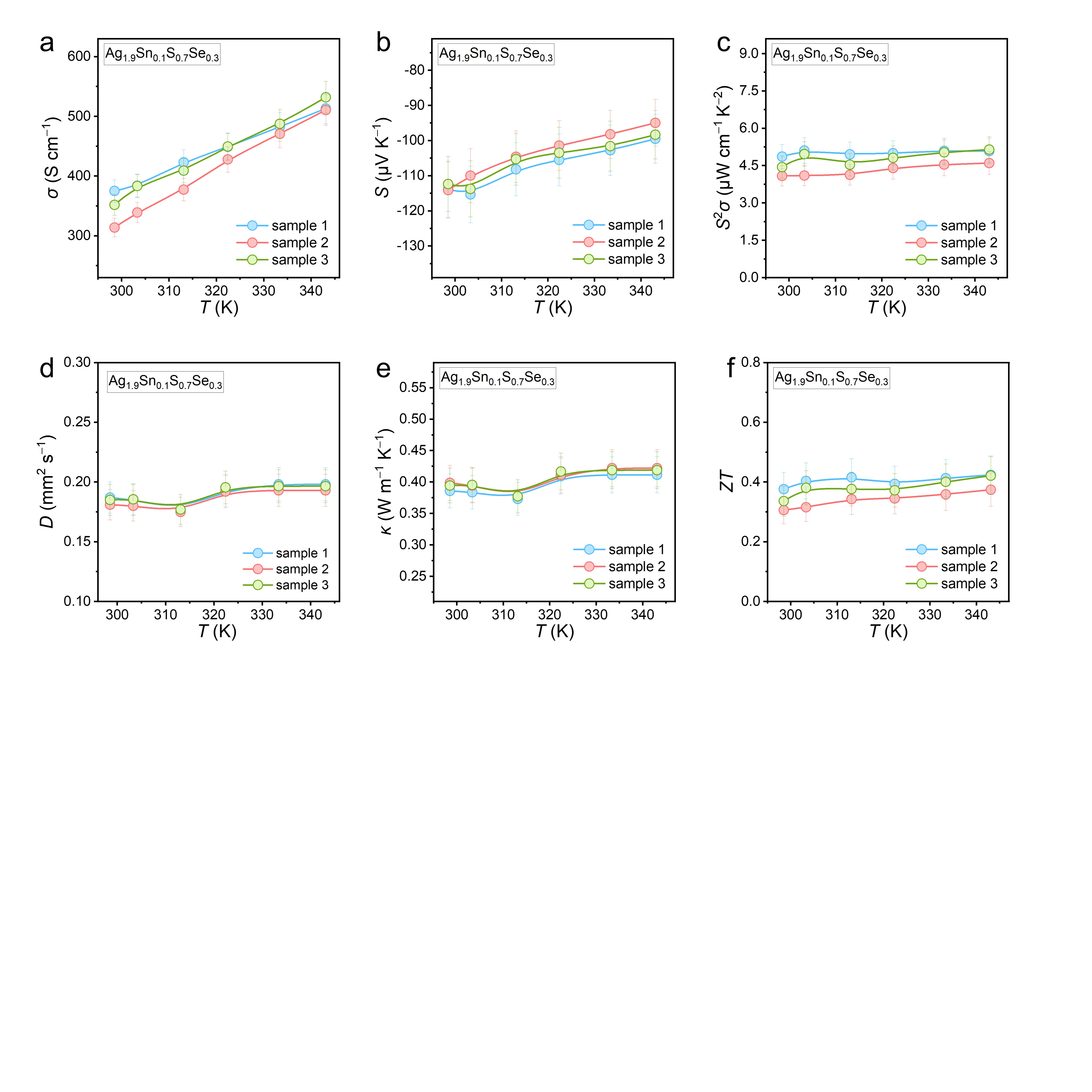
**

**Figure S10.** The temperature-dependent (a) electrical conductivity (*σ*), (b) Seebeck coefficient (*S*), (c) power factor (*S*^2^*σ*), (d) *D*, (e) *κ*, and (f) *ZT* value of different batches of Ag_1.9_Sn_0.1_S_0.7_Se_0.3_ samples.

**
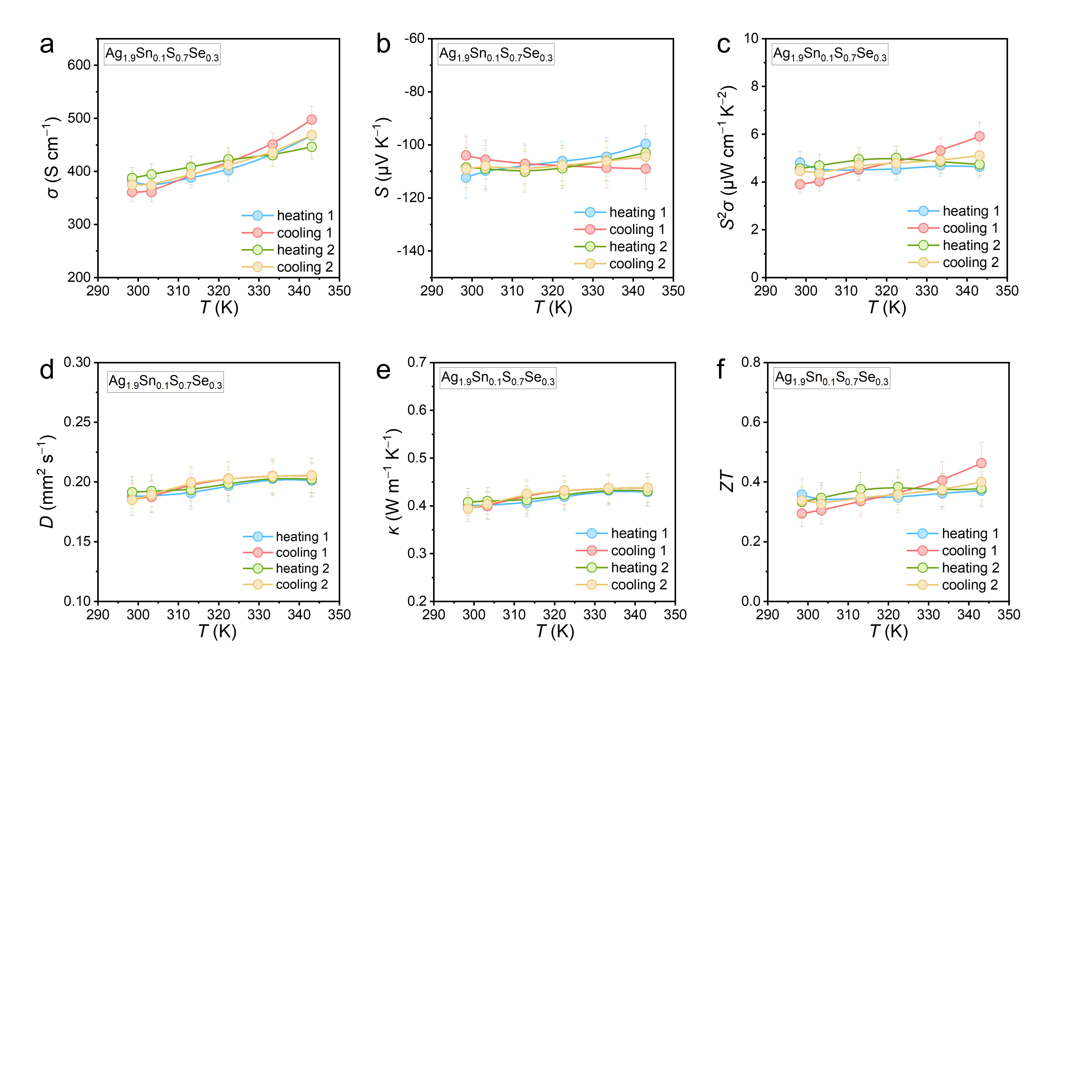
**

**Figure S11**. The temperature-dependent (a) *σ*, (b) *S*, (c) *S*^2^*σ*, (d) *D*, (e) *κ*, and (f) *ZT* value of Ag_1.9_Sn_0.1_S_0.7_Se_0.3_ sample under different heating and cooling circles.

**
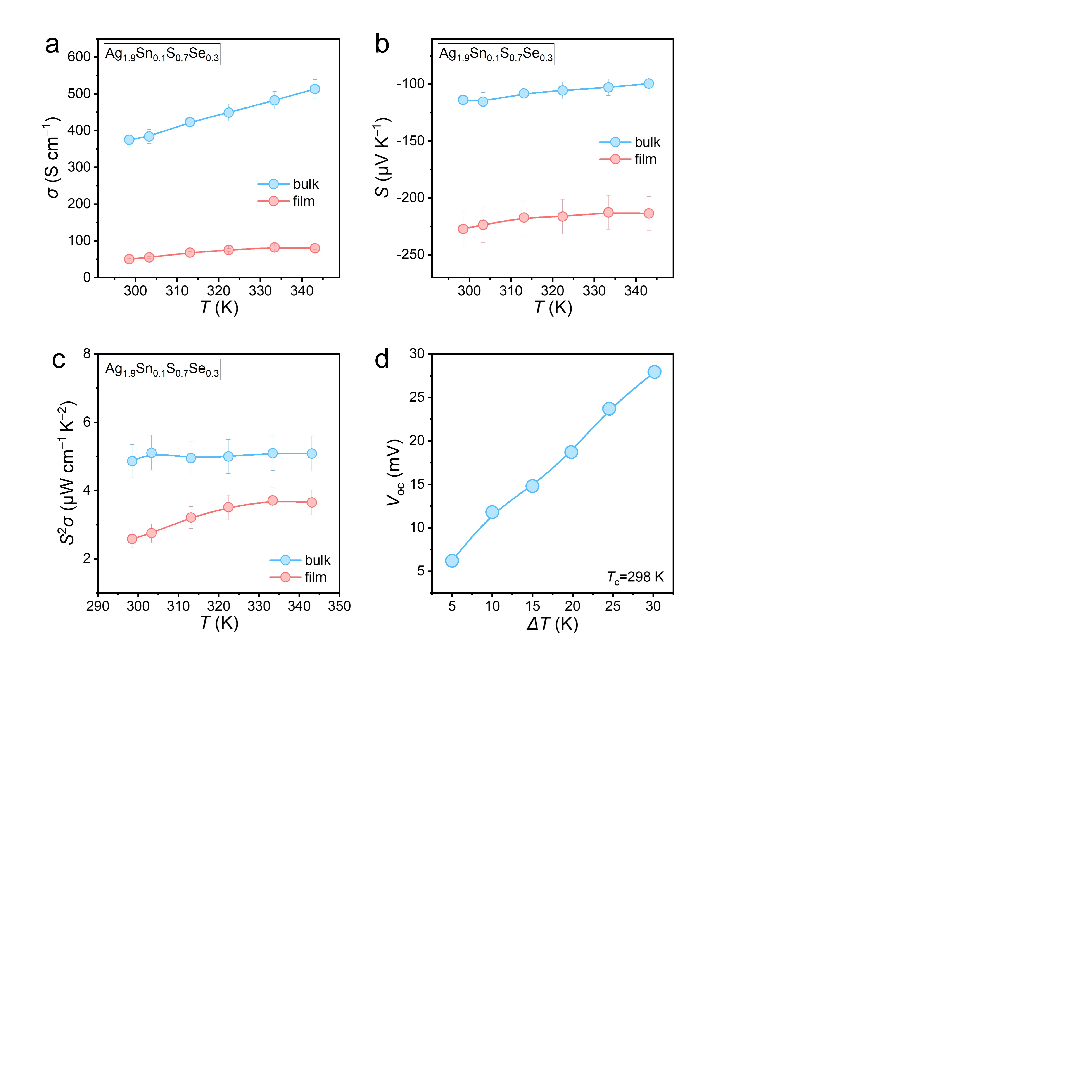
**

**Figure S12.** The temperature-dependent (a) *σ*, (b) *S*, and (c) *S*^2^*σ* of Ag_1.9_Sn_0.1_S_0.7_Se_0.3_ bulk and film. (d) The current (*I*) dependent open circuit voltage (*V*_oc_) of the device under different temperature differences (*ΔT*).

**References**

[1] H. Wu, X.-L. Shi, W.-D. Liu, M. Li, H. Gao, W. Zhou, Z. Shao, Y. Wang, Q. Liu, Z.-G. Chen, *Chem. Eng. J.* **2021**, *425,* 130668.

[2] G. Kresse, J. Hafner, *Phys. Rev. B* **1994**, *49,* 14251-14269.

[3] G. Kresse, J. Hafner, *Phys. Rev. B* **1993**, *47,* 558-561.

[4] G. Kresse, J. Furthmüller, *Comp. Mater. Sci.* **1996**, *6,* 15-50.

[5] G. Kresse, J. Hafner, *J. Phys. Condens. Mat.* **1994**, *6,* 8245-8257.

[6] G. Kresse, J. Furthmüller, *Phys. Rev. B* **1996**, *54,* 11169-11186.

[7] G. Kresse, D. Joubert, *Phys. Rev. B* **1999**, *59,* 1758-1775.

[8] J.P. Perdew, K. Burke, M. Ernzerhof, *Phys. Rev. Lett.* **1996**, *77,* 3865-3868.

[9] W. Setyawan, S. Curtarolo, *Comp. Mater. Sci.* **2010**, *49,* 299-312.

[10] F. Tran, P. Blaha, *Phys. Rev. Lett.* **2009**, *102,* 226401.

[11] M. Cococcioni, S. de Gironcoli, *Phys. Rev. B* **2005**, *71,* 035105.

[12] V.I. Anisimov, J. Zaanen, O.K. Andersen, *Phys. Rev. B* **1991**, *44,* 943-954.

[13] R. Nelson, C. Ertural, J. George, V.L. Deringer, G. Hautier, R. Dronskowski, *J. Comput. Chem.* **2020**, *41,* 1931-1940.

[14] X.L. Shi, K. Zheng, M. Hong, W.D. Liu, R. Moshwan, Y. Wang, X.-L. Qu, Z.G. Chen, J. Zou, *Chem. Sci.* **2018**, *9,* 7376-7389.

[15] X. Shi, A. Wu, T. Feng, K. Zheng, W. Liu, Q. Sun, M. Hong, S.T. Pantelides, Z.G. Chen, J. Zou, *Adv. Energy Mater.* **2019**, *9,* 1803242.

[16] X. Shi, A. Wu, W. Liu, R. Moshwan, Y. Wang, Z.-G. Chen, J. Zou, *ACS Nano* **2018**, *12,* 11417-11425.

[17] M. Jin, X.-L. Shi, T. Feng, W. Liu, H. Feng, S.T. Pantelides, J. Jiang, Y. Chen, Y. Du, J. Zou, Z.-G. Chen, *ACS Appl. Mater. Interfaces* **2019**, *11,* 8051-8059.

[18] Y. Chang, Z. Li, P. Luo, W. Qian, J. Zhang, J. Luo, *Adv. Funct. Mater.* **2023**, 2310016.

[19] J. Liang, T. Wang, P. Qiu, S. Yang, C. Ming, H. Chen, Q. Song, K. Zhao, T.-R. Wei, D. Ren, Y.-Y. Sun, X. Shi, J. He, L. Chen, *Energy Environ. Sci.* **2019**, *12,* 2983-2990.

[20] T. Wang, H.-Y. Chen, P.-F. Qiu, X. Shi, L.-D. Chen, *Acta Phys. Sin-ch. Ed.* **2019**, *68,* 090201.

[21] H. Hu, Y. Wang, C. Fu, X. Zhao, T. Zhu, *The Innovation* **2022**, *3,* 100341.

[22] J. Liu, T. Xing, Z. Gao, J. Liang, L. Peng, J. Xiao, P. Qiu, X. Shi, L. Chen, *Appl. Phys. Lett.* **2021**, *119,* 121905.

[23] S. Yang, Z. Gao, P. Qiu, J. Liang, T.-R. Wei, T. Deng, J. Xiao, X. Shi, L. Chen, *Adv. Mater.* **2021**, *33,* 2007681.
